# Intranasal vaccination of hamsters with a Newcastle disease virus vector expressing the S1 subunit protects animals against SARS-CoV-2 disease

**DOI:** 10.1038/s41598-022-13560-z

**Published:** 2022-06-20

**Authors:** Manolo Fernández Díaz, Katherine Calderón, Aldo Rojas-Neyra, Vikram N. Vakharia, Ricardo Choque-Guevara, Angela Montalvan-Avalos, Astrid Poma-Acevedo, Dora Rios-Matos, Andres Agurto-Arteaga, Maria de Grecia Cauti-Mendoza, Norma Perez-Martinez, Gisela Isasi-Rivas, Luis Tataje-Lavanda, Yacory Sernaque-Aguilar, Freddy Ygnacio, Manuel Criollo-Orozco, Edison Huaccachi-Gonzalez, Elmer Delgado-Ccancce, Doris Villanueva-Pérez, Ricardo Montesinos-Millán, Kristel Gutiérrez-Manchay, Katherinne Pauyac-Antezana, Ingrid Ramirez-Ortiz, Stefany Quiñones-Garcia, Yudith Cauna-Orocollo, Katherine Vallejos-Sánchez, Angela Rios-Angulo, Dennis Núñez-Fernández, Mario I. Salguedo-Bohorquez, Julio Ticona, Manolo Fernández-Sánchez, Eliana Icochea, Luis A. Guevara-Sarmiento, Mirko Zimic, COVID-19 Working Group in Perú, Andres Agurto-Arteaga, Ricardo Antiparra, Manuel Ardiles-Reyes, Katherine Calderón, Yudith Cauna-Orocollo, Maria de Grecia Cauti-Mendoza, Naer Chipana-Flores, Ricardo Choque-Guevara, Xiomara Chunga-Girón, Manuel Criollo-Orozco, Lewis De La Cruz, Elmer Delgado-Ccancce, Nicolás E. Delgado-Pease, Christian Elugo-Guevara, Manolo Fernández-Díaz, Manolo Fernández- Sánchez, Luis A. Guevara-Sarmiento, Kristel Gutiérrez-Manchay, Oscar Heredia-Almeyda, Edison Huaccachi Gonzalez, Pedro Huerta-Roque, Eliana Icochea, Gisela Isasi-Rivas, Gabriel Jiménez-Avalos, Romina A. Juscamaita-Bartra, Abraham Licla-Inca, Angela Montalvan-Avalos, Ricardo Montesinos-Millán, Dennis Núñez-Fernández, Adiana Ochoa-Ortiz, Gustavo E. Olivos-Ramirez, Erika Páucar-Montoro, Katherinne Pauyac-Antezana, Jose L. Perez-Martinez, Norma Perez-Martinez, Astrid Poma-Acevedo, Stefany Quiñones-Garcia, Ingrid Ramirez-Ortiz, Daniel Ramos-Sono, Angela Rios-Angulo, Dora Rios-Matos, Aldo Rojas-Neyra, Yomara K. Romero, Mario I. Salguedo-Bohorquez, Yacory Sernaque-Aguilar, Patricia Sheen, Luis F. Soto, Luis Tataje-Lavanda, Julio Ticona, Vikram N. Vakharia, Katherine Vallejos-Sánchez, A. Paula Vargas-Ruiz, Doris Villanueva-Pérez, Renzo G. Villena, Freddy Ygnacio, Mirko Zimic

**Affiliations:** 1 Farmacológicos Veterinarios S.A.C. FARVET, Panamericana Sur N° 766 Km 198.5. Chincha Alta, Ica, Peru; 2 Institute of Marine and Environmental Technology, University of Maryland Baltimore County, Baltimore, USA

**Keywords:** SARS-CoV-2, Live attenuated vaccines, Viral infection

## Abstract

The coronavirus disease-19 (COVID-19) pandemic has already claimed millions of lives and remains one of the major catastrophes in the recorded history. While mitigation and control strategies provide short term solutions, vaccines play critical roles in long term control of the disease. Recent emergence of potentially vaccine-resistant and novel variants necessitated testing and deployment of novel technologies that are safe, effective, stable, easy to administer, and inexpensive to produce. Here we developed three recombinant Newcastle disease virus (rNDV) vectored vaccines and assessed their immunogenicity, safety, and protective efficacy against severe acute respiratory syndrome coronavirus 2 (SARS-CoV-2) in mice and hamsters. Intranasal administration of rNDV-based vaccine candidates elicited high levels of neutralizing antibodies. Importantly, the nasally administrated vaccine prevented lung damage, and significantly reduced viral load in the respiratory tract of vaccinated animal which was compounded by profound humoral immune responses. Taken together, the presented NDV-based vaccine candidates fully protected animals against SARS-CoV-2 challenge and warrants evaluation in a Phase I human clinical trial as a promising tool in the fight against COVID-19.

## Introduction

The severe acute respiratory syndrome coronavirus 2 (SARS-CoV-2), causative agent of coronavirus disease-19 (COVID-19), recognizes the angiotensin-2 converting enzyme (ACE-2) which are present on the surface of several human cell types including pneumocytes. The surface glycosylated spike (S) protein is mainly attributed to the receptor binding, promoting endocytosis and resulting in the entry of the virus^1,2^. Out of the two distinctive subunits of S protein (S1 and S2), the most distal end of the S1 subunit is the receptor binding domain (RBD), which interacts with ACE-2 receptor through the receptor binding motif (RBM) to initiate the infection and entry process^3^. It’s been demonstrated that neutralizing antibodies from COVID-19 convalescent patients are commonly directed towards specific epitopes in the S1 and S2 subunits^4–6^. Our earlier understanding of coronaviruses such as the severe acute respiratory syndrome coronavirus (SARS-CoV) and Middle Eastern respiratory syndrome coronavirus (MERS-CoV) has helped to identify potential SARS-CoV-2 vaccine candidates targeting the S protein for its known immunogenicity^7–10^. The S protein of SARS-CoV-2 has also been found to carry potential B and T lymphocyte protective epitopes with the potential for vaccine candidate^11,12^. Beside full-length S protein (pre-fusion or post-fusion), the S1 and RBD domains are considered important vaccine targets^13^ and have been the focus of vaccine development.

However, the amino acid sequences of S1/RBD are found to be under a selection pressure, seeking a greater affinity for ACE-2^14–18^ or escape from neutralization by antibodies against S1 of SARS-CoV-2^19,20^. Different strategies have been applied for the development of vaccines against SARS-CoV-2, seeking safety, effectiveness and protection against the virus, including vaccines based on inactivated virus, on mRNA, and using viral vectors^21–24^.

Newcastle disease virus (NDV), the causative agent of the Newcastle disease (ND), has been used as a viral vector for the expression of diverse antigens from myriads of animal and human pathogens^25–27^. The NDV, also known as *Avian orthoavulavirus 1,* is a member of the *Paramyxoviridae* family^28^ and carry a single-stranded, negative-sense RNA virus with a genome size of approximately 15.2 kb^29,30^. The NDV encodes six structural proteins in the order of nucleocapsid protein (NP), phosphoprotein (P), matrix protein (M), fusion protein (F), haemagglutinin–neuraminidase protein (HN), and the large protein (L), which is a viral polymerase^29,30^.

The NDV can be divided into three groups according to their virulence in poultry: velogenic, mesogenic, and lentogenic^29^. The LaSota strain of NDV is lentogenic (apathogenic), and is routinely used as a live attenuated NDV vaccine in poultry. Importantly, it grows to a high titer in embryonated chicken eggs, induces strong humoral and cellular immune responses, and can be administered via the nasal route^30^ due to its receptor abundance in upper respiratory tracts. It has been demonstrated that NDV does not pose a threat to human health, and waste majority of the human population do not exhibit pre-existing immunity^26,31^. Owing to ectopic expression and cytoplasmic replication nature, the NDV-vectored vaccines induce mucosal immune response at the respiratory tract, and do not recombine with host DNA during replication^32^.

NDV has been used as a vector for vaccine development since the late 1990s in different animal hosts. The efficiency of vaccines based on this vector has been demonstrated against respiratory viruses, such as infectious bronchitis and avian reovirus in chickens, SARS-CoV in monkeys, and MERS-CoV in camels^33–36^. These studies have demonstrated that it is feasible to generate NDV expressing the S protein from other coronaviruses, such as SARS-CoV and MERS-CoV, which conferred strong immunogenicity and protection in mice and non-human primates^35,36^. Recently, NDV has been proposed as a potential vector for a vaccine against SARS-CoV-2.

Other studies, based on NDV-vectored vaccines expressing the complete spike S protein, that was administered either by the nasal or the intramuscular route, evaluated in hamsters and mice induced high immune response, eliciting Immunoglobulin G (IgG) and Immunoglobulin A (IgA) antibodies. Animals were highly protected against challenge with SARS-CoV-2. Infection, inflammation, or any pathological lesion was prevented in lung tissues, while corporal weight and physical mobility was maintained normal. The viral load in lungs of challenged animals, compared with the control, was reduced, as well as virus shedding in nasal turbinates and lungs in hamsters and mice^37–40^.

In this study, we describe the design and evaluation of an intranasal NDV-vectored vaccine in hamsters challenged with SARS-CoV-2. In the present study we demonstrated the same levels of efficacy of protection and safety. In contrast, our NDV-vectored vaccine expressed the S1 subunit and RBD domain, and were administered through the nasal route.

## Results

In this study we developed and evaluated three different intranasal vaccine formulations, including an NDV presenting the RBD domain, an NDV presenting the S1 subunit, and a combination of both the RBD domain and S1 subunits. These vaccines were administered through the nasal route in two doses. Safety and efficacy was confirmed in hamsters challenged with SARS-CoV-2. NDV-vectored expressing S1 showed a high protection against challenge in contrast to NDV-vectored expressing RBD.

### Development and characterization of recombinant NDV expressing SARS-CoV-2 RBD and S1 antigens

#### Generation of rNDVs expressing individually RBD and S1 subunit genes of SARS-CoV-2

Vero-E6 cells were co-transfected with full-length plasmid cDNA of constructs pFLC-LS1-HN-RBD and pFLC-LS1-S1-F together with three supporting plasmids encoding for the NP, P, and L proteins of NDV, essential for the replication of NDV. At 72 h post-transfection, the cells showed several visible plaques with typical cytopathic effect (CPE) from NDV, demonstrating the successful rescue of both recombinant viruses. Supernatants collected five days after transfection were injected into allantoic cavities of 9-day-old embryonated SPF eggs. The allantoic fluid was harvested four days after inoculation and analyzed by HA using chicken red blood cells. We found positive HA titers ranging from 2 to 2048.

**Figure 1:**
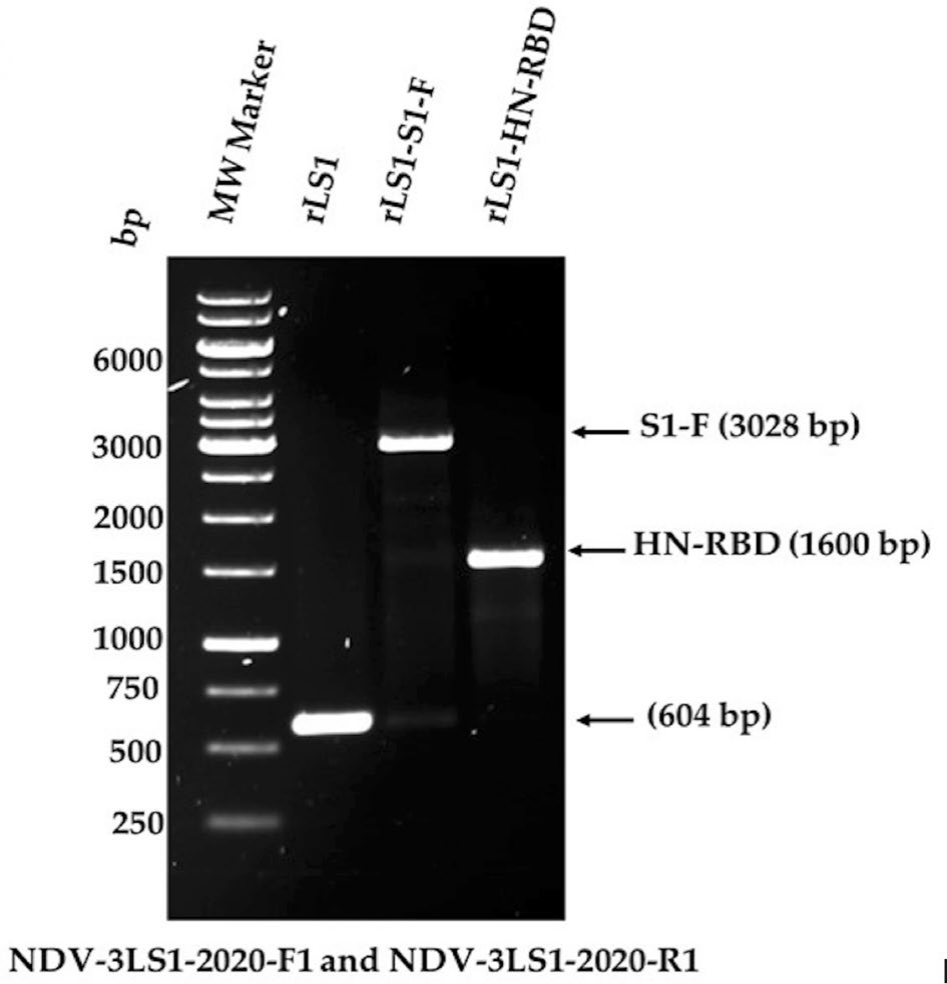
The insertion of the expression cassette into the non-coding region between the P/M genes of NDV genome was verified by RT-PCR using the primers NDV-3LS1-2020-F1 and NDV-3LS1-2020-R1.

The presence of the HN-RBD and S1-F expression cassettes inserted into the non-coding region between the P and M genes of the NDV genome were verified by RT-PCR, yielding fragments of 1600 and 3028 base pairs (bp), respectively. These fragments were subsequently amplified and sequenced using primers (NDV-3LS1-2020-F1 and NDV-3LS1-2020-R1), demonstrating proper insertion into the NDV genome (Fig. 1). These new recombinant NDV viruses were named rLS1-HN-RBD and rLS1-S1-F, respectively.

#### Expression of the SARS-CoV-2 Proteins from rLS1-HN-RBD and rLS1-S1-F Viruses

**Figure 2:**
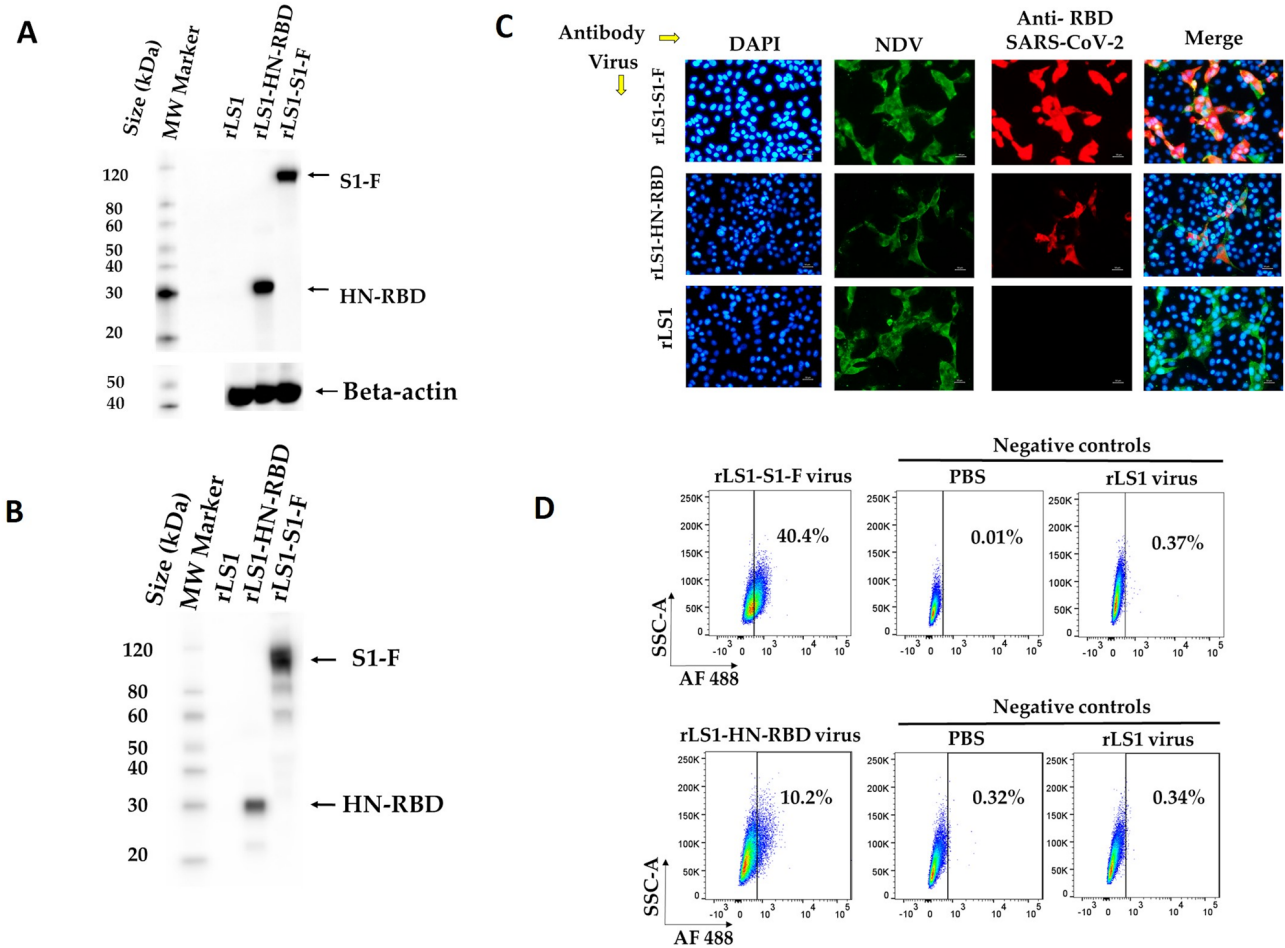
Expression of SARS-CoV-2 RBD and S1 proteins in infected Vero-E6 cells and NDV particles. (**A**) Western blot detection for the HN-RBD and S1-F proteins expression. Vero-E6 cells were infected with the rLS1, rLS1-HN-RBD, and rLS1-S1-F viruses at an MOI of 1.0. After 48 hpi, the cells were lysed and analyzed by Western blotting. (**B**) To verify the incorporation of the HN-RBD and S1-F proteins into rLS1-HN-RBD, and rLS1-S1-F viruses, the viral particles in allantoic fluid of infected SPF chicken embryonated eggs with the recombinant viruses and rLS1, was concentrated by ultracentrifugation, and partially purified on a 25% sucrose cushion. Western blot analysis was carried out using partially purified viruses and lysate from infected cells, using a rabbit antibody specific to SARS-CoV-2 RBD protein and Anti-rabbit IgG conjugated to HRP. The beta-actin protein was used as a loading control in lysate cells. The black arrow indicates the expected protein band. The gels shown were run under identical conditions. (**C**) Vero-E6 cells infected with the rLS1, rLS1-HN-RBD, and rLS1-S1-F at an MOI of 0.5. After 48 hpi, the expression of RBD and S1 proteins was detected by immunofluorescence assay using a rabbit antibody specific to SARS-CoV-2 RBD protein, and a Donkey Anti-rabbit IgG H&L-Alexa Fluor 594. Therefore, the NDV was detected using a chicken antiserum specific to the NDV, and a Goat Anti-chicken IgY H&L-Alexa Fluor 488. Cell nuclei were stained with DAPI. A scale bar of 50-µm. Image magnification 200×. (**D**) Detection of S1 or RBD proteins on the viral surface of rLS1-S1-F and rLS1-HN-RBD viruses’ attachment to Vero-E6 cells was performed in two independent experiments. The cells were incubated with purified viruses rLS1-HN-RBD or rLS1-S1-F, for 30 min. Subsequently, the cells were labeled with rabbit monoclonal antibody anti-SARS-CoV-2 S1 as the primary antibody, followed by secondary antibody goat anti-rabbit IgG Alexa Fluor 488. The cells were then analyzed by a flow cytometer. The percentage of positive cells indicates the detection of S1 or RBD proteins on the viral surface of viruses bound to Vero-E6 and is shown in the dot plot for rLS1-S1-F virus and rLS1-HN-RBD virus; including negative controls for each assay determined by cells incubated with DPBS or rLS1 virus.

In infected cell lysates, a protein band was detected for each of the recombinant viruses, with a molecular mass of ~ 90 kDa (S1-F) for the rLS1-S1-F virus and a band of ~ 30 kDa (HN-RBD) for the rLS1-HN-RBD virus (Fig. 2A), respectively. Likewise, the same result was obtained with the purified recombinant virus (Fig. 2B), confirming that the S1-F or HN-RBD proteins were incorporated into the viral particles of rLS1-S1-F and rLS1-HN-RBD viruses, respectively. As expected, these protein bands were not detected in the rLS1-infected cells or in purified viral particles from the rLS1 virus. Multiple exposure images for Fig. 2A,B are provided in the supplementary material (supplementary Figures 1, 2, respectively). An rLS1-F-RBD, expressing RBD fused with TM and CT domains from F gene of NDV was generated.

However, after inserting this construct into the NDV genome, we were unable to detect the expression of the RBD (expected size of 27.9 kDa) after evaluating 4 clones by Western blot (supplementary Figure 3).

The expression of the SARS-CoV-2 RBD and S1 subunits was detected in Vero-E6 cells infected with rLS1-HN-RBD, and rLS1-S1-F by IFA. The RBD and S1 subunit expression was not detected in cells infected with the rLS1 virus. The NDV protein expression was detected using a chicken antiserum specific to NDV, and a Goat Anti-Chicken IgY H&L-Alexa Fluor 488 in Vero-E6 cells infected with rLS1, rLS1-HN-RBD, and rLS1-S1-F viruses (Fig. 2C). Detection of SARS-CoV-2 S1 subunit or RBD on the viral surface of rLS1-S1-F and rLS1-HN-RBD viruses bound to Vero-E6 cells was confirmed by flow cytometry in two independent experiments (Fig. 2D). For the rLS1-S1-F virus, 40.4% positive cells were detected, for the rLS1-HN-RBD virus 10.2% positive cells were detected, and for the rLS1 virus up to 0.37% positive cells were detected. A higher percentage of cells was detected in the rLS1-S1-F virus than the rLS1-HN-RBD virus.

#### Genetic stability of the rLS1-HN-RBD and rLS1-S1-F viruses

**Figure 3:**
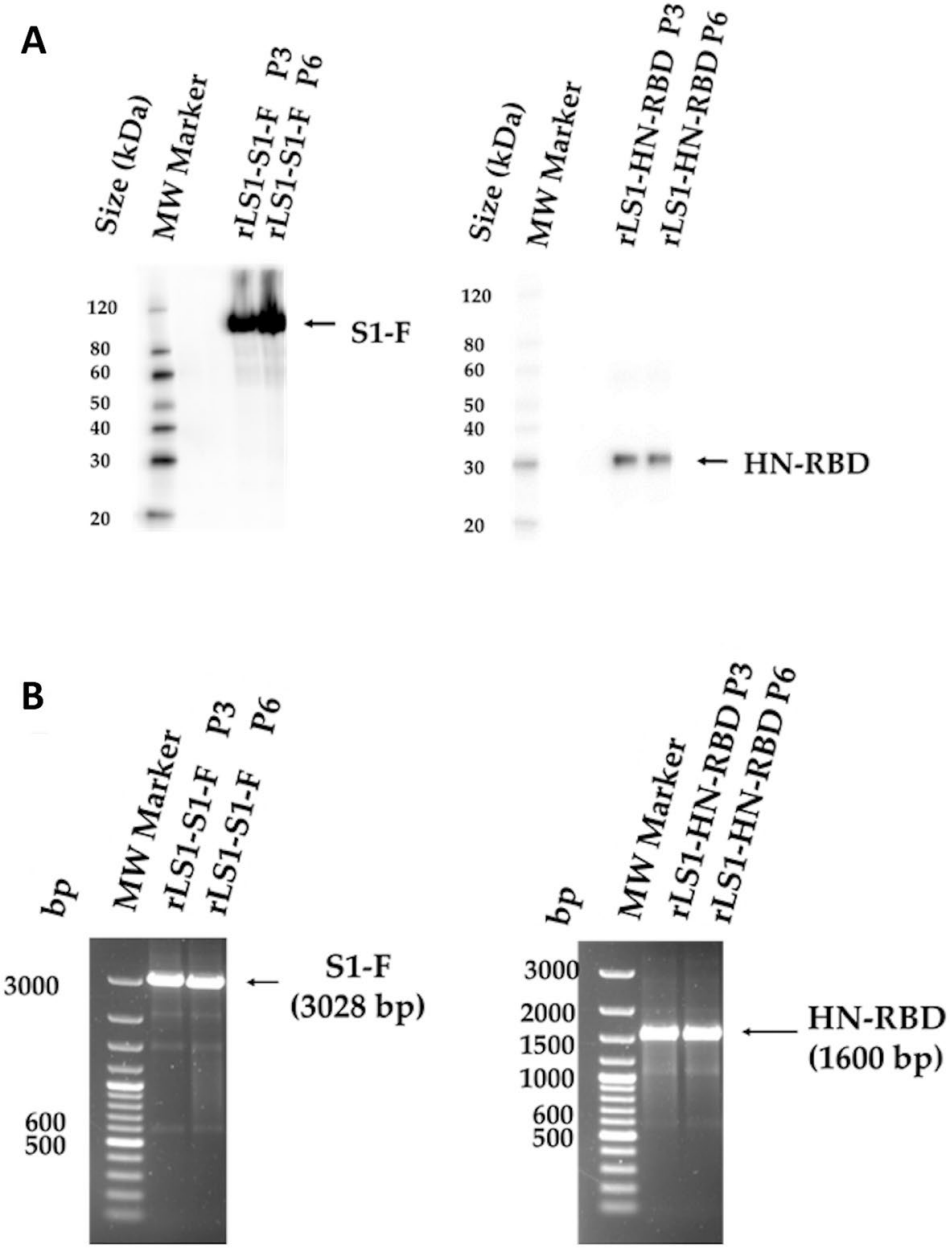
Genetic stability of the recombinants. The genetic stability of the rLS1-HN-RBD, and rLS1-S1-F viruses was evaluated at the 3rd and 6th passages by (**A**) Western blot analysis using a rabbit polyclonal antibody specific to SARS-CoV-2 RBD protein, and (**B**) RT-PCR using the primers NDV-3LS1-2020-F1 and NDV-3LS1-2020-R1 to amplify the complete inserts. P3: 3rd passage, P6: 6th passage. Black arrow indicates the expected molecular mass.

In order to demonstrate the stability of the exogenous gene expression, the RT-PCR was conducted at the 3rd and 6th passage which showed presence of an expected size of the gene inserts. Western blot analysis confirmed the expression of the S1-F and HN-RBD proteins during these passages, suggesting genetic stability and correct expression of the inserts in the recombinant viral genome (Fig. 3A,B) until sixth passage.

#### Stability of the lyophilized vaccine

Before the lyophilization, the titer in plaque assay of the vaccine was 8.12 ± 0.03 log_10_ PFU/mL determined by plaque assay and the HA titer was 2^10^.

**Figure 4:**
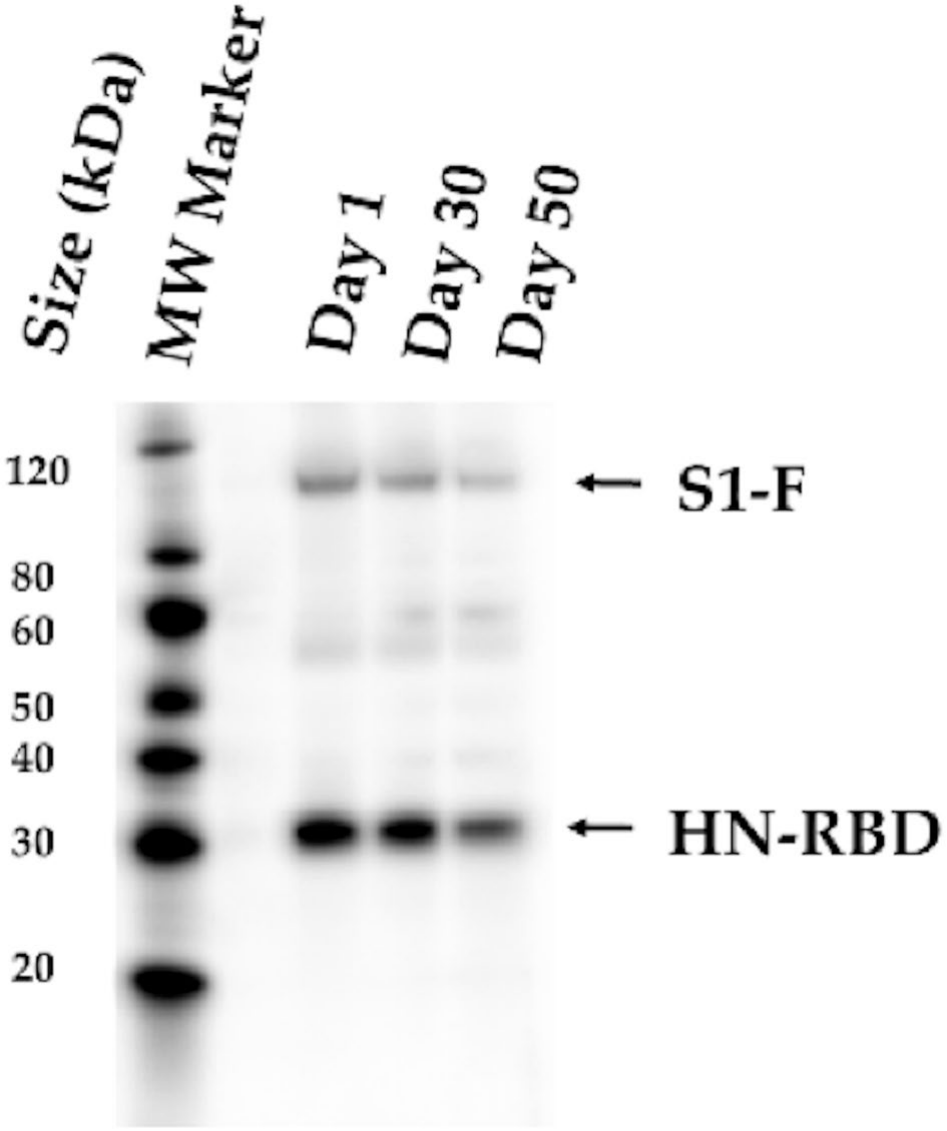
Stability of the lyophilized NDV vaccine. The expression of S1-F and HN-RBD proteins in Vero-E6 cells infected with the lyophilized NDV vaccine was confirmed at day 1, 30, and 50 days post-lyophilization by Western blot assay using a rabbit polyclonal antibody specific to SARS-CoV-2 RBD protein. Black arrow indicates the expected protein band.

After lyophilization, and after storing the vaccine variants at 4 °C, the vaccine was reconstituted. The titers after reconstitution resulted stable at days 1, 30, and 50 with 8.37 ± 0.06, 7.69 ± 0.24, and 8.24 ± 0.05 log_10_ PFU/mL. Similarly, the HA titers were stable after each of the three storage periods (2^10^). The expression of S1-F and HN-RBD proteins in Vero-E6 cells infected with the lyophilized NDV vaccine was confirmed after reconstitution at days 1, 30, and 50 post-lyophilization by Western blot (Fig. 4).

### Immunogenicity in hamsters

#### Intranasal vaccination elicits specific antibodies against S protein and neutralizing antibodies against SARS-CoV-2 in hamsters

**Figure 5:**
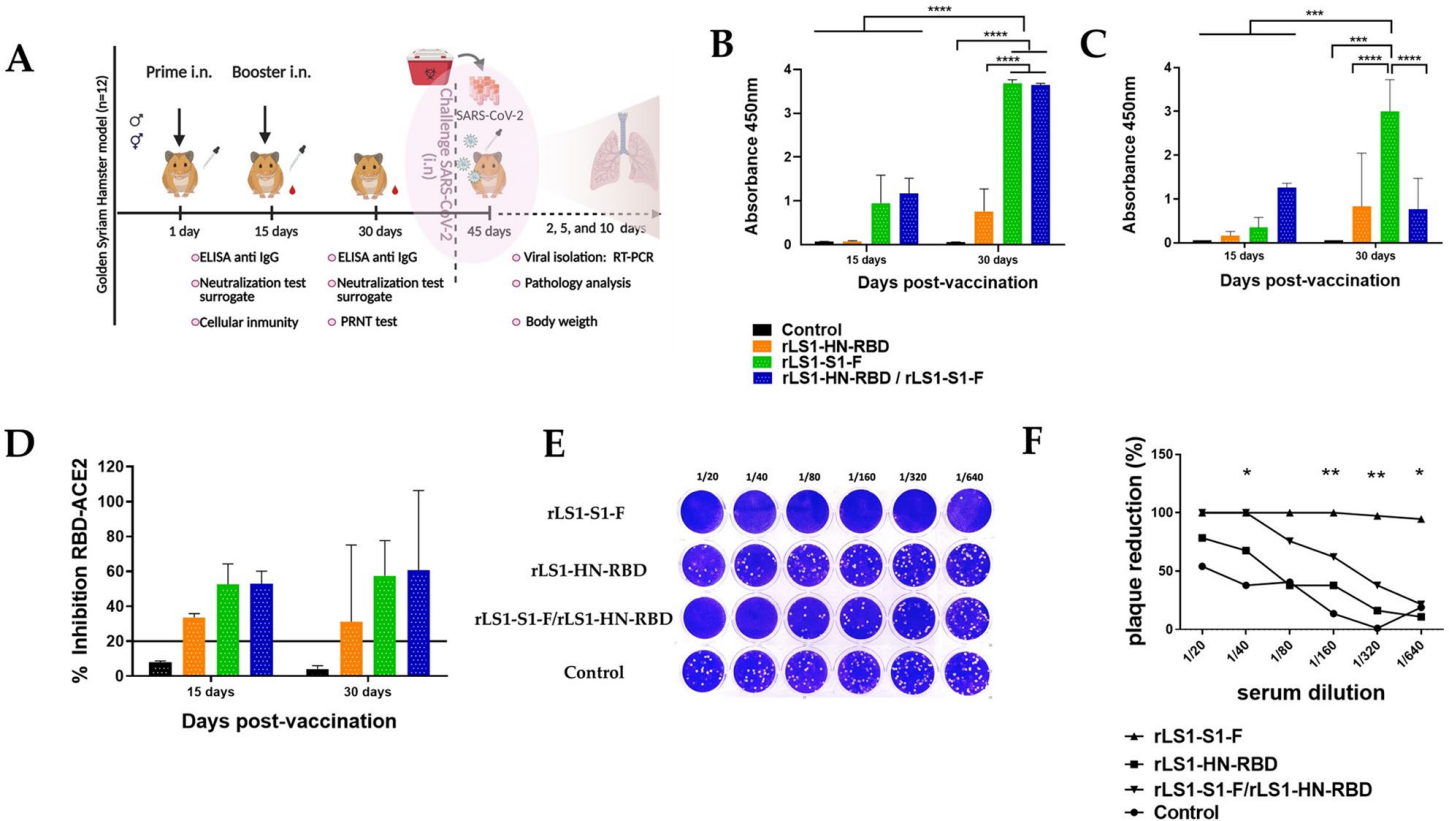
The intranasal vaccine elicits specific antibodies against RBD and S1 protein and neutralizing antibodies against SARS-CoV-2 in hamsters. (**A**) Immunization regimen. To evaluate the immunogenicity of the NDV vaccines, five- week-old female and male golden Syrian hamsters were used in this study. The hamsters were randomly divided into four groups. The hamsters were vaccinated by intranasal route with live NDV vaccine, following a prime-boost- regimen with a two-week interval. Group 1 received rLS1-HN-RBD (*n* = 12), Group 2 received the rLS1-S1-F (*n *= 12), Group 3 received the mixture of rLS1-HN-RBD/rLS1-S1-F (*n* = 12), and Group 4 was the non-vaccinated control group (*n* = 12) One booster immunization with the same concentration of each vaccine was applied in all vaccinated groups at the second week. (**B**) ELISA assay to measure SARS-CoV-2 RBD-specific serum IgG antibody, and (**C**) S1 subunit-specific serum IgG antibody. Sera from hamsters at pre-boost and 15 days after boost were evaluated. SARS-CoV-2 RBD purified recombinant protein was used for ELISA. The cutoff was set at 0.06. (**D**). Immunized hamsters were bled pre-boost and 15 days after boost. All sera were isolated by low-speed centrifugation. Serum samples were processed to evaluate the neutralizing antibody titers against SARS-CoV-2 RBD protein using the sVNT. The positive cut-off and negative cut-off for SARS-CoV-2 neutralizing antibody detection were interpreted as the inhibition rate. The cut-off interpretation of results: result positive ≥ 20% (neutralizing antibody detected), result negative < 20% (neutralizing antibody not detectable. (**E**) Figure depicts titers of PRNT of SARS-CoV-2 on Vero cells with pooled serum from hamsters immunized with rLS1-S1-F, rLS1-HN-RBD, and the mixture of both. (**F**) Plaque reduction (%) curves using pooled serum from the different groups of hamsters. Two-way ANOVA and Tukey’s post hoc were performed. *P < 0.05; **P < 0.01; ***P < 0.001; ****P < 0.0001.

Fifteen days after prime immunization, the hamster groups immunized with the live rLS1-HN-RBD, rLS1-S1-F, and the combined rLS1-HN-RBD/rLS1-S1-F vaccines developed specific serum IgG antibodies against S1 and RBD (Fig. 5A). At 15 days post-boost (30 days post-immunization), there was a significant increase in the titers of serum IgG antibodies. The control group did not induce SARS-CoV-2 S1 or RBD-specific serum IgG antibodies. Importantly, immunization with rLS1-S1-F and the combination of vaccines rLS1-HN-RBD/rLS1-S1-F induced significantly higher level of serum IgG antibodies specific to S1 and RBD at 15 days after the boost compared to rLS1-HN-RBD (Fig. 5B,C).

Additionally, neutralization assays using the sVNT indicated that the sera from groups immunized with rLS1-HN-RBD, rLS1-S1-F, and rLS1-HN-RBD/rLS1-S1-F developed neutralizing antibodies specific to RBD protein at 15 days post-immunization and 15 days post-boost.

However, the sera collected from hamsters vaccinated with rLS1-S1-F and rLS1-HN-RBD/rLS1-S1-F showed a percentage of inhibition of the RBD-ACE-2 binding greater than 50%, whereas the rLS1-HN-RBD group only showed 30% inhibition up to 15 days post-boost. Sera from the control group remained below 20% inhibitory up to 15 days post-boost and did not show neutralizing antibodies against the RBD protein (Fig. 5D).

Pooled serum from hamsters vaccinated with the rLS1-S1-F virus showed a high plaque reduction titer in the neutralization assay at 15 days post-boost, retaining 100% of this capacity even at higher dilutions of serum (1/160). The combined rLS1-HN-RBD/rLS1-S1-F vaccine showed a lower titer of viral plaque reduction (1/40) than rLS1-S1-F. On the other hand, the. rLS1-HN-RBD had no effect on plaque reduction (Fig. 5E,F).

#### Cytokines quantification by ELISA

Immunization of hamsters did not induce a significant increase in serum levels of IL-2 or IFNγ, evaluated by quantitative ELISA. Although non-significant, an increasing trend of levels of IL-2 with rLS1-HN-RBD (*P* = 0.55), rLS1-S1-F (*P* = 0.07) or rLS1-HN-RBD/rLS1-S1-F (*P* = 0.07) was observed. However, one of the individuals vaccinated with rLS1-HN-RBD/rLS1-S1-F had high levels of circulating IL-2 production. Levels of IFNγ were not increased significantly with rLS1-HN-RBD (*P* = 0.08), rLS1-S1-F (*P* = 1.00) or rLS1-HN-RBD/rLS1-S1-F (*P* = 0.56) (Supplementary Figure 5). The levels of IL-4 and IL-10 remained below the lower limit of detection, and the TNFα values were low and were detectable only in serum from animals immunized with rLS1-S1-F and rLS1-HN-RBD/rLS1-S1-F (Supplementary Table I).

#### Cytokines quantification by qPCR

No significant difference in cytokine gene expression was observed for any of the vaccines used: for rLS1-HN-RBD (IFNγ 1.2-fold, *P* = 1.00, TNFα 1.48-fold, *P* = 0.16, IL-10 1.7-fold, *P* = 0.16), rLS1-S1-F (IFNγ 1.16-fold, *P* = 0.16, TNFα 1.48-fold, *P* = 0.14, IL-10 *P* = 0.16) or rLS1-HN-RBD/rLS1-S1-F (IFNγ 0.81-fold, *P* = 0.48, TNFα 1.12-fold, *P* = 0.48, IL-10 0.90, *P* = 0.48). Although non-significant, trends are observed (Supplementary Figure 5).

### Efficacy of the vaccines against SARS-CoV-2 challenge

**Figure 6:**
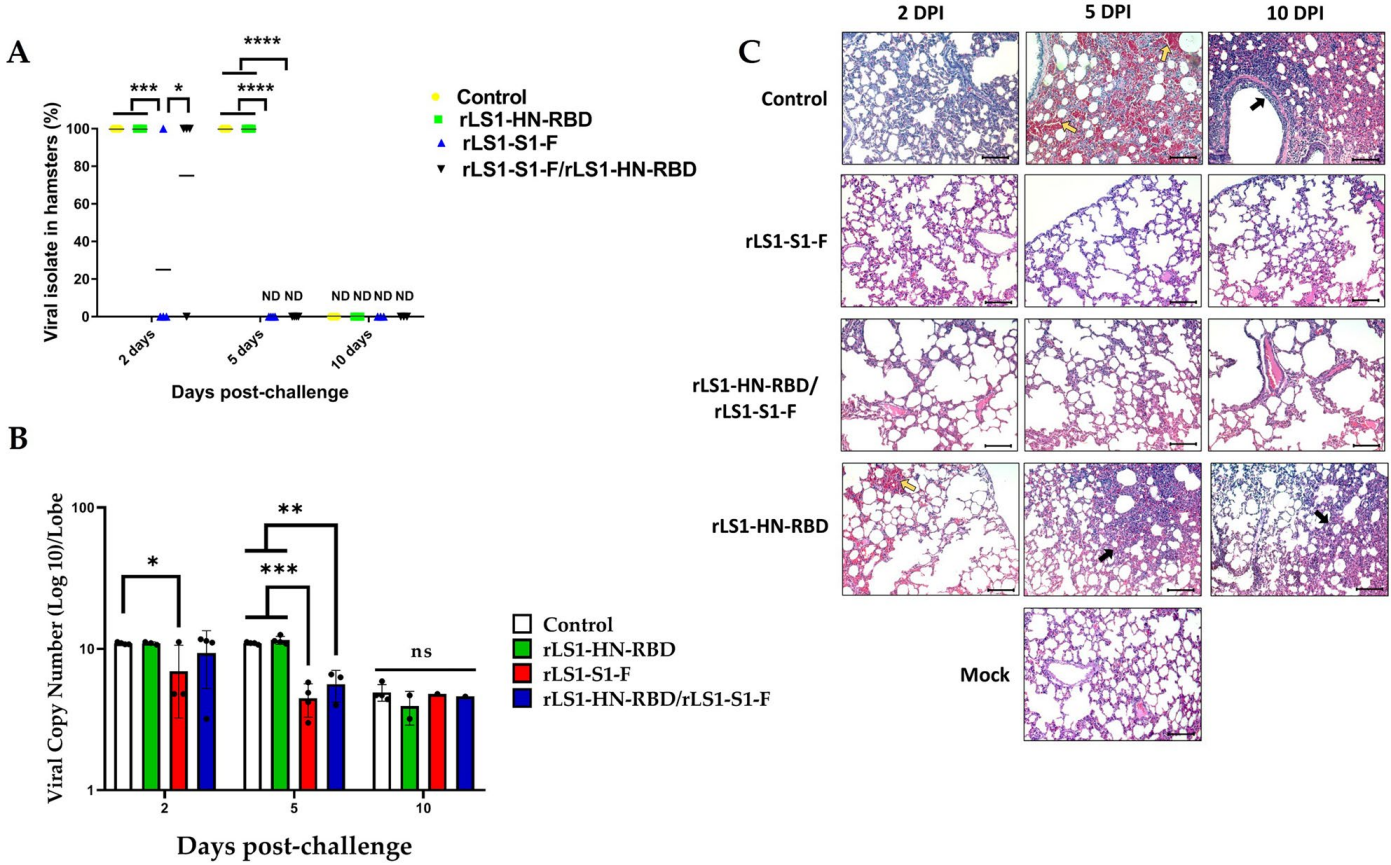
Efficacy of live NDV vaccines against SARS-CoV-2 infection in hamsters. Golden Syrian hamster groups vaccinated with rLS1-S1-F, rLS1-HN-RBD, the mixture rLS1-S1-F/rLS1-HN-RBD, or non-vaccinated (positive control) were challenged 30 days after the boost with SARS-CoV-2, along with a mock group that was non-vaccinated nor challenged (negative control). (**A**) Viral isolation (% of animals that were positive for SARS-CoV-2) was done from the lung of each hamster group (*n* = 4) at days 2, 5, and 10 post-challenge. Two-way ANOVA and Tukey’s post hoc were performed. *P < 0.05; **P < 0.01; ***P < 0.001; ****P < 0.0001. (**B**) Detection by RT-qPCR of SARS-CoV-2 in culture supernatant of Vero cells, inoculated with lung homogenates of immunized and challenged hamster groups. The data show a significant difference in the viral copy number *P < 0.05; **P < 0.01; ***P < 0.001; ****P < 0.0001. ns, not significant. (**C**) Lung histopathology of each hamster group (n = 4) was euthanized at different days post-infection (DPI). Hemorrhagic and infiltrated areas are indicated by a yellow and black arrow, respectively. Image amplitude: 20×. Scale-bar: 100 µm.

On days 5 and 10 post-challenge, no SARS-CoV-2 was isolated from the lung tissue of hamsters vaccinated with rLS1-S1-F and the mix rLS1-S1-F/rLS1-HN-RBD vaccine. The vaccinated and challenged animals showed a negative IFA result at days 5 and 10 post-challenge. The rLS1-HN-RBD vaccine alone did not demonstrate sufficient neutralizing capacity to prevent the infection. In this group, the virus could be isolated at day 5 post-challenge and confirmed by a positive IFA in the lung tissue (Fig. 6A, Supplementary Tables II, III). Among the non-vaccinated hamsters, SARS-CoV-2 was isolated on days 2 and 5 post-challenge, but by day 10, no virus could be detected.

Viral copy quantification by RT-qPCR obtained from hamster lung homogenates confirmed the presence of a high viral copy number (10.97 ± 0.17 log_10_) in unimmunized (control) hamsters and hamsters immunized with the rLS1-HN-RBD (10.96 ± 0.24 log_10_) and rLS1-HN-RBD/rLS1-S1-F (9.36 ± 4.09 log_10_) vaccines at day 2 post-challenge. Animals immunized with rLS1-S1-F showed a lower viral copy number significantly (6.92 ± 3.70 log_10_, *P* < 0.05). At day 5 post-challenge, the viral load from the isolate was maintained in the control group (10.99 ± 0.24 log_10_) and in hamsters immunized with rLS1-HN-RBD (11.54 ± 0.76 log_10_), however, in hamsters immunized with rLS1-S1-F (4.50 ± 1.17 log_10_, *P* ≤ 0.0003) and rLS1-HN-RBD/rLS1-S1-F (5.65 ± 1.41 log_10_, *P* ≤ 0.0059) the viral load decreased significantly (Fig. 6B). On day 10 post-challenge all of the groups analyzed, including the control, presented a low viral copy number (3.94–4.93 log_10_) (Fig. 6B), probably due to the presence of residual RNA, since we did not detect any cytopathic effect on Vero cell culture.

The histopathological changes of the hamster lungs were monitored during the SARS-CoV-2 challenge. The unvaccinated (control) group demonstrated pathological signs of the disease, starting with interstitial pneumonia at 2 dpc, evolving into hemorrhagic pneumonia at 5 dpc, and ending up in severe bronchopneumonia, characterized by a thickening in the parenchyma wall, moderate to severe infiltration of macrophage and lymphocyte inflammatory cells, and in bronchiolar lumen, and loss of alveolar architecture. The groups immunized with rLS1-S1-F and rLS1-HN-RBD/rLS1-S1-F vaccines did not show visible lesions, maintaining characteristics of the lung tissue similar to those seen in the unchallenged group at each of the evaluated points. Although the group immunized with rLS1-HN-RBD developed pathological changes in the lung, those were milder than those seen in the unvaccinated group, showing moderate pneumonia 2 dpc and ending in a moderate to severe pneumonia at 10 days (Fig. 6C).

**Figure 7:**
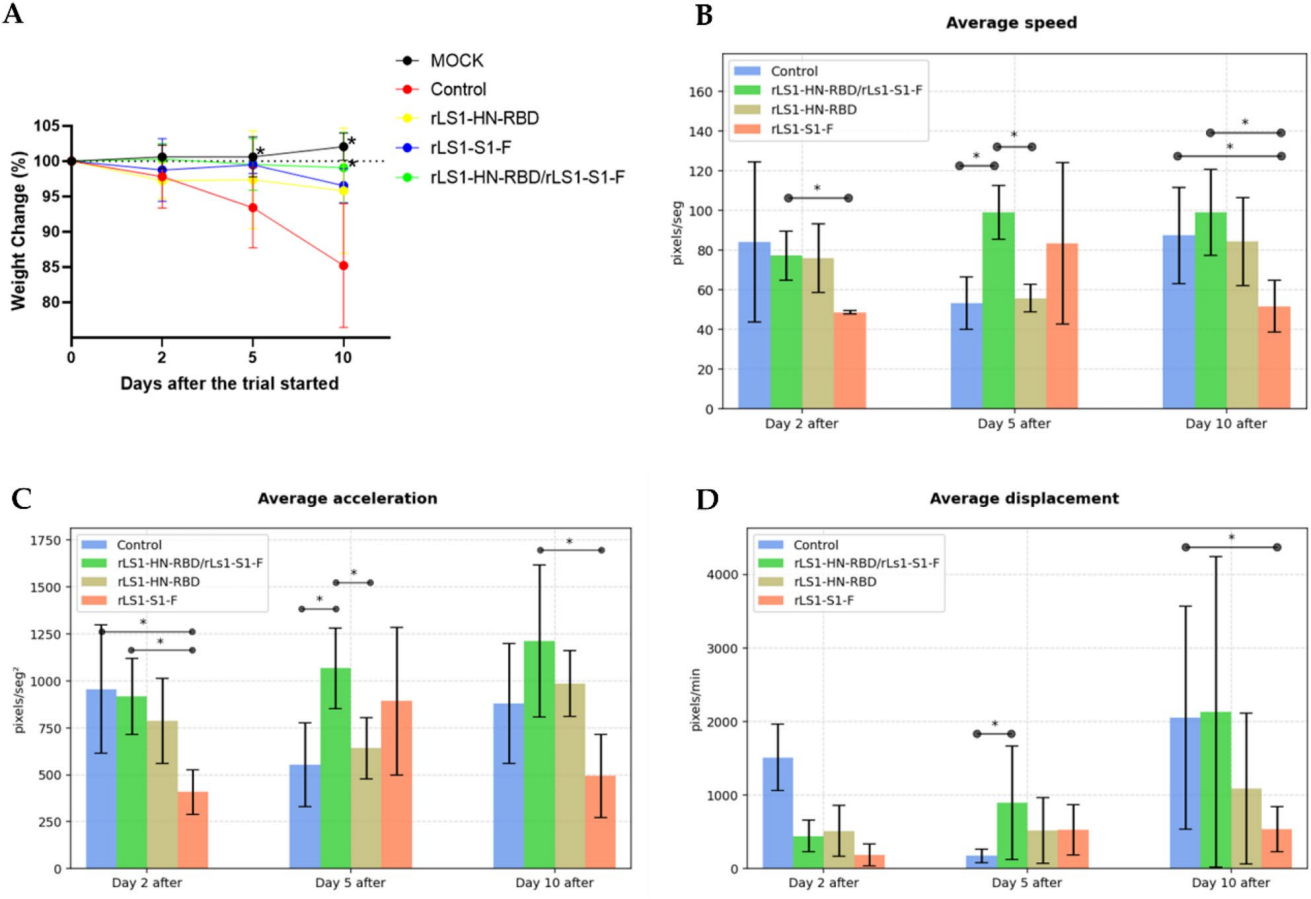
Body weight and mobility analysis of SARS-CoV-2 challenged golden Syrian hamsters. (**A**) Changes in body weight (percent weight change compared to day 0) of hamsters inoculated with SARS-CoV-2 and Mock group, at days 2, 5, and 10 post-challenged. Mobility assessment results shown (**B**) average velocity, (**C**) average acceleration, and (**D**) average displacement. Mean ± SD are shown. Asterisks indicate that results were statistically significant compared to the control group (*P < 0.05).

Unvaccinated animals belonging to the unchallenged control group showed an average percentage variation in body weight of no more than 3% over the 10 days of analysis. Unvaccinated animals that were infected with SARS-CoV-2 showed significant weight loss, with an average reduction in body weight of over 5% by day 5 and over 10–25% on day 10 dpc. There were no statistically significant differences body weight between the vaccinated and unvaccinated-unchallenged hamsters on days 2 and 5, but there was a significant difference between the challenged control group and the rLS1-HN-RBD/rLS1-S1-F vaccinated group at day 10 (Fig. 7A).

Assessments of the animals’ average speed, acceleration, and displacement confirmed that hamsters vaccinated with the combined rLS1-S1-F/rLS1-HN-RBD vaccine were the most active on day 5 (time at which symptoms appear) and day 10 (time at which symptoms disappear) post-challenge. Animals vaccinated with the combined rLS1-S1-F/rLS1-HN-RBD vaccine showed the greatest recovery in terms of mobility (Fig. 7B–D).

## Discussion

In this study, we developed two recombinant NDV-vectored nasal vaccine candidates expressing the SARS-CoV-2 S1 subunit and RBD antigens. Three different intranasal vaccine formulations were evaluated including an NDV presenting the RBD domain, an NDV presenting the S1 subunit, and a combination of both NDV-vectored with the RBD domain and S1 subunit. Efficacy of protection assessment in hamsters showed that the strongest immunity and protection were elicited by the NDV-vectored with the S1 subunit, followed by the mix combining the RBD domain and S1 subunit, as evidenced by the lack of any damage to the lung cells, considerably reduced viral load and viral viability, as compared to the non-vaccinated group. Surprisingly, the RBD domain vaccine did not show any evidence of protection. There are at least two possible explanations that are not necessarily mutually exclusive. First, the S1 subunit may include protective epitopes in addition to those present in the RBD domain, for which neutralizing antibodies could contribute in interfering with the S-ACE-2 interaction perhaps at a distant steric level. This possibility is supported by a previous study that showed that the S1 subunit contains neutralizing epitopes which were not found in the RBD^7^. Second, the RBD present in the rLS1-HN-RBD vaccine does not reach a folding close enough to the 3D structure of the biologically active RBD when it is in the SARS-CoV-2 viral particle. Therefore, it is possible that conformational B epitopes may be playing a more important role than linear T epitopes in the protective immune response. Recent studies that evaluated a similar vaccine candidate vectored in NDV exposing the complete S antigen (S1 and S2 domains) demonstrated protection of hamsters in a challenge assay^38,39^.

Of note, hamsters from the non-vaccinated group that were challenged with SARS-CoV-2 positive virus cultures at 2 and 5 dpc were negative at day 10. These results are in agreement with previous studies which showed that viral load is reduced to undetectable levels by 8 days after infection in the hamster model^41,42^. Along with the in vitro results, the IFA also confirmed that the S1 subunit vaccine, followed by the combined rLS1-HN-RBD/rLS1-S1-F vaccine, induced the strongest protective responses. This was evidenced by failure to isolate virus from the lung tissue at 5 dpc. At 2 dpc, only half of the animals vaccinated with the S1 subunit vaccine had virus isolated, and these had a negative IFA. This suggests that neutralization of SARS-CoV-2 is likely to be occurring between 2 and 3 dpc in the hamster model.

Histopathological evaluation of lung from non-vaccinated hamsters after the challenge showed severe pathological signs of the disease, beginning with interstitial pneumonia 2 dpc, and ending in severe bronchopneumonia, as well as the loss of alveolar architecture. In contrast, the groups immunized with the S1 subunit and RBD domain/S1 subunit were protected, showing almost intact alveoli and respiratory capillaries, without evidence of an inflammatory reaction. However, the animals vaccinated with the RBD domain vaccine developed pathological lesions, although with a lesser hemorrhagic degree than the non-vaccinated control group, with pneumonia at 2 dpc and ending in a moderate to severe pneumonia at 10 dpc. This is an encouraging result that needs to be verified in human clinical trials.

After intranasal challenge, the animals were tested for presence and viability of SARS-CoV-2 in the lungs, at days 2, 5 and 10, time at which animals were sacrificed and necropsied. At day 5 after the challenge, animals vaccinated with rLS1-S1-F and rLS1-S1-F/rLS1-HN-RBD did not show the presence of viable virus in the lungs, in contrast to the control and the vaccinated with rLS1-HN-RBD animals. However, since we did not measure the virus shedding from the nasal cavity, we can’t speculate that virus shedding could be stopped in the vaccinated animals. Further studies are needed to better understand this fact.

To evaluate the immune response, vaccinated animals were bled two weeks before the challenge. It was important to reduce the stress in the animals that may temporarily impair the immune response at the moment of the challenge. Although other studies use a shorter time, we thought that two weeks is more than enough to have the animals recover from the stress caused during the bleeding. However, we recognize this is a limitation of our study, as we do not have a measurement of the immunity level at the exact moment of the challenge and therefore our measurements don’t fully reflect immune responses at that time.

When measuring RBD specific and S1 specific antibodies, we observe that the animals vaccinated with rLS1-HN-RBD or rLS1-S1-F did show a boosting from day 15 to day 30 post-vaccination, for both RBD and S1 specific antibodies. However, in the animals vaccinated with the mixture rLS1-HN-RBD/rLS1-S1-F, there is a boosting for the RBD specific antibodies, but there is a lack of boosting for the S1 specific antibodies. Unfortunately, we do not have evidence for a clear explanation of this pattern. We can speculate that the lack of boosting for the S1 antibodies among the animals vaccinated with rLS1-HN-RBD/rLS1-S1-F, could be associated with a potential interaction between the two vaccines, in which the mixture of both, results in a dominance of the RBD antigen resulting in a lack of boosting of non-RBD antibodies (i.e. S1 antibodies different than RBD antibodies). Further studies are required to clarify this issue.

In this report, we evaluated the mobility pattern of animals as an objective and quantitative indicator of their health status. Using recorded videos and computational tools for pattern analysis and digital tracking, we measured the average velocity, the average acceleration, and the average displacement of the animals in their cages at 2, 5, and 10 dpc. The results showed that at 5 dpc, the animals from the unvaccinated control group had a reduced average displacement, velocity, and acceleration compared to the vaccinated animals. This marked difference was not clearly observed at 2 dpc, confirming that at that time point, the infected animals may have been relatively asymptomatic. This result agrees with other findings that show clearance of the virus at 10 dpc^41,42^.

A promising result that suggests strong protection of the rLS1-S1-F vaccine is the neutralization capacity of the vaccinated hamsters’ sera against the SARS-CoV-2 virus at 30 days after immunization in the PRNT. In contrast, animals vaccinated with rLS1-HN-RBD showed marginal sero-neutralization capacity. This result is consistent with similar findings reported in a recent study showing that an intranasal NDV-vectored live attenuated SARS-CoV-2 vaccine induced high level of humoral immunity in a relatively short time, as well as the production of mucosal IgA^39^. A mucosal antibody response is considered important against infections that use the respiratory tract as a route of entry, making the respiratory tract mucosa the first line of defense against this infection. Currently, we are completing an assay to evaluate the presence of anti-S1/RBD mucosal IgA antibodies in mice vaccinated with rLS1-S1-F. Preliminary data show evidence of IgA production in bone marrow cells (data not shown). Further studies are required to confirm this finding.

Other recent studies, evaluated similar NDV-vectored vaccines in hamsters and mice. In contrast to the vaccines described here, others used the complete spike S protein^37–40^. In these reports, the NDV-vectored vaccine was administered through the nasal or the intramuscular route. These vaccines elicited high immune response, with specific IgG and IgA antibodies. Efficacy evaluation with SARS-CoV-2 challenge after immunization, confirmed that neither Infection, inflammation, nor any pathological lesion was found in lung tissues. The viral load in lungs of challenged animals, compared with the control, was reduced, as well as virus shedding in nasal turbinates and lungs in hamsters and mice. Body weight and physical mobility was not altered^37–40^. Our results evaluating the safety and efficacy of the proposed vaccine are very similar to the ones described previously in the literature. Although it is recognized that the nasal route has advantages, as for the elicitation of IgA mucosal antibodies, if the elicited immune response is much lower, the advantage can be lost.

Attenuated NDV is most commonly used as a vaccine around the world against ND infection, with doses of median tissue culture infectious dose (TCID_50_) ranging from 10^4^ to 10^5^, which is administered to animals by oral, nasal, ocular, or spray delivery^43–45^. Since NDV is restricted to the respiratory tract, it is not detected in other organs or blood, therefore replication in humans is expected to be limited and benign^46–48^. Rarely, humans exposed to mesogenic NDV have been observed to develop conjunctivitis, laryngitis, or flu-like symptoms that disappear within 1 to 2 days^49^. Thus, vaccinators and caretakers who are frequently exposed to the NDV have not reported any side effects to date^50–52^. Of note, safety and toxicity test were conducted with doses there were not produced under strict good manufacturing practice (GMP) certification, meaning that any adverse effect will be over-estimated. Since the trials were conducted with non GMP doses of the vaccine means that any adverse events will be over-projected, and lower rates would be expected in an evaluation of the vaccine at doses produced under GMP conditions.

The development of the NDV-vectored vaccine candidate presented here includes a final lyophilization step. This confers stability: the vaccine can be stored at 4 °C for several months without losing more than 5% of its activity, similar to other lyophilized vaccines^53^. The fact that the rLS1-S1-F vaccine is administered through the nasal route gives it a further advantage in simplifying the logistical requirements for immunizations. There is no need for an army of vaccinators or large numbers of syringes. It is possible that doses of rLS1-S1-F vaccine could be delivered in 500-dose vials with a manual trigger-activated dispenser system that uses individual disposable tips. In this way, a nasal vaccine could be delivered in large-scale campaigns in remote rural communities with great ease.

The COVID-19 pandemic has the potential to become endemic, and if this were to happen vaccines would need to be routinely administered with some frequency^54,55^. The SARS-CoV-2 in recent months has shown an intense level of mutations in the viral antigens used in the various vaccines currently available. These mutations have been selected naturally, in the face of the immunological pressure exerted by individuals cured of COVID-19. Thus, mutations have now been identified that may give the virus the ability to escape acquired immunity (immune resistance), and this may lead to a surge in cases of SARS-CoV-2 reinfection. These same naturally selected mutations have also been selected in vitro, under immunological selection pressures using convalescent serum neutralizing antibodies. This suggests a high possibility that vaccines based on circulating S1 antigen in the early 2020's may be compromised to some degree in their level of effectiveness against new SARS-CoV-2 variants.

Therefore, the most efficient way to deal with the COVID-19 pandemic will be to use vaccines customized for specific geographic areas, based on the distribution of circulating variants over a certain period of time, and which can be produced and administered promptly. The S1 subunit vaccine can be upgraded and carry a vaccine antigen corresponding to a more relevant strain in a relatively short time. NDV can be transformed within 30-45 days, and a master cryobank generated to start producing updated vaccine batches. It is therefore important to have permanent epidemiological surveillance programs to identify any variation in the distribution of circulating strains in a region of interest.

*In conclusion*, we have demonstrated that S1 subunit vaccine candidate shows promise in preclinical studies. This vaccine candidate was shown to be safe and immunogenic, and provided strong protection against a SARS-CoV-2 challenge. Clinical trials are now needed to evaluate its safety and efficacy in humans.

## Methods

### Ethics statements

Animal research was conducted following relevant guidelines and regulations. All experimental protocols were approved by the Bioethics Committee of the Universidad Nacional Hermilio Valdizán, Huánuco, Peru. The study was carried out in compliance with the ARRIVE guidelines.

SARS-CoV-2 (28549) virus used in the challenge was provided by the National Institute of Health (INS), Lima, Peru, in accordance with relevant guidelines and regulations. The isolation of the SARS-CoV-2 virus was approved by the General Direction of the Public Health Centre of the INS.

### Animals

One hundred male and female Golden Syrian hamsters (*Mesocricetus auratus*) aged 4-5 weeks were obtained from the Peruvian National Institute of Health (INS). For the in-vivo assay, all hamsters were transferred and acclimatized to the Animal Biosafety Level 3 (BSL-3) facility for 1 week. There, they were vaccinated with NDV-vectored SARS-CoV-2 vaccine and later challenged with live SARS-CoV-2.

### Development and characterization of recombinant NDV expressing SARS-CoV-2 RBD and S1 antigens

#### Cell Culture

African green monkey kidney cells, clone E6 (Vero-E6, ATCC^®^ CRL-1586^TM^) and DF-1 cells (derived from Chicken Fibroblast), were maintained in Dulbecco’s modified Eagle’s medium (DMEM), supplemented with 5% heat-inactivated fetal bovine serum (FBS) (HyClone^TM^ GE Healthcare Life Science, USA). Vero cells (Vero 81, ATCC^®^ CCL-81^TM^) were grown in Eagle’s Minimum Essential Medium (EMEM) supplemented with 10% FBS, 100 IU/mL of penicillin, and 100 µg/mL streptomycin. All cell lines were cultivated at 37 °C in an atmosphere of 5% CO_2_.

#### Plasmid construction

Our vaccine candidates are based on the recombinant lentogenic NDV strain LaSota, which was designated the rLS1 virus. The design and construction of a pFLC-LS1 plasmid (19,319 nucleotides (nt)) containing the full-length genome of an infectious NDV clone, and the three support plasmids containing the N, P, and L genes (pCI-N, pCI-P, and pCI-L, respectively) have been previously described^56^. This NDV-based system is protected under a Peruvian patent 001179-2014/DIN.

The genetic sequences of the RBD and the S1 subunit of the S protein correspond to the SARS-CoV-2 Wuhan-Hu-1 strain isolate from China *(*GenBank accession no. MN908947.3). In the case of S1, due to the larger size of its sequence, and to facilitate the replication and production of the vaccine in the embryonated eggs, the codon usage was optimized for *Gallus gallus*.

**Figure 8:**
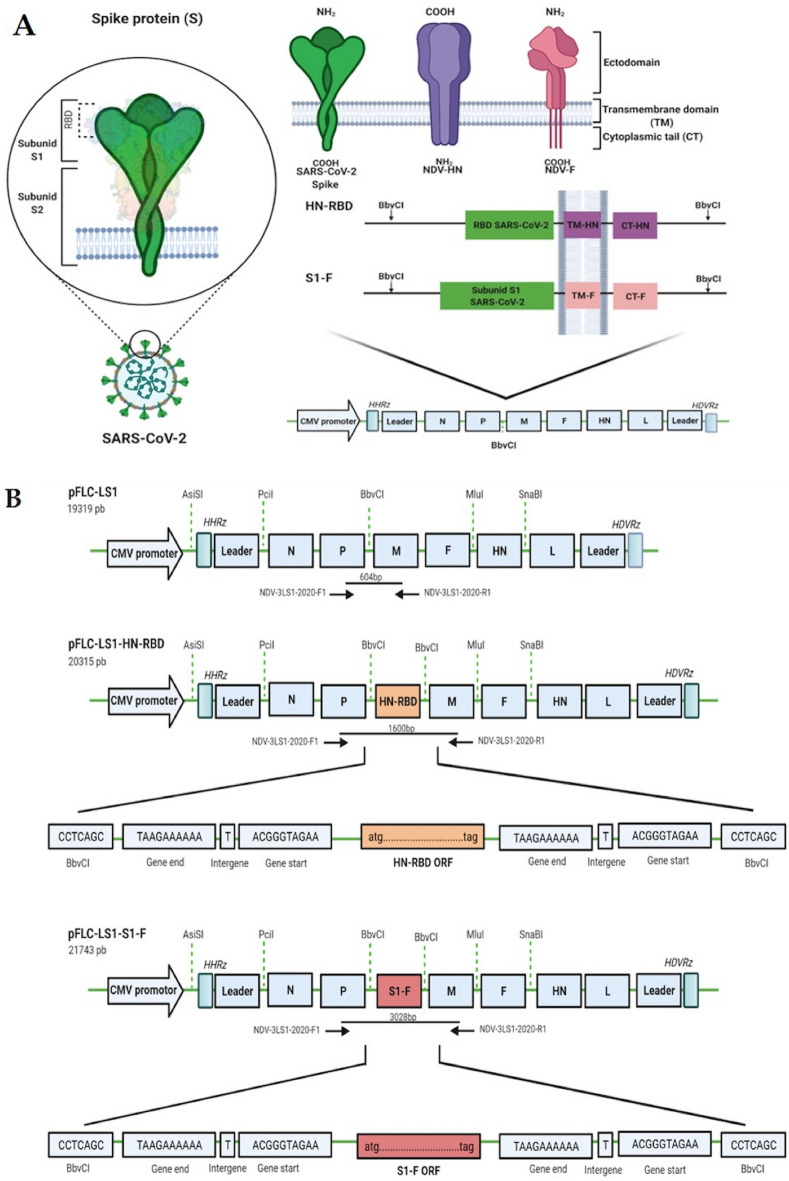
The strategy used for the generation of the recombinant NDVs expressing SARS-CoV-2 RBD and S1. (**A**) The schematic representation of the strategy of construction of the recombinant NDVs. Two transcriptional cassettes were designed for expressing RBD and S1: 1) HN-RBD was fused with the complete transmembrane domain (TM) and the cytoplasmic tail (CT) of the haemagglutinin–neuraminidase (HN) gene, 2) S1-F was fused with the TM/CT of the fusion (F) gene from the full-length pFLC-LS1. (**B**) The full-length anti-genome of NDV strain LaSota clone (pFLC-LS1) was used as a backbone clone, the pFLC-LS1-HN-RBD and pFLC-LS1-S1-F were generated from cassettes expressing RBD and S1 genes inserted into NDV genome under control of transcriptional gene end (GE) and gene start (GS) signals. The names, position, and direction of the primers used are shown with arrows (blacks) indicating the size products.

To improve the incorporation of RBD and S1 into the NDV virion, we designed two cassettes. First, the HN-RBD transcriptional cassette (1013 nt) contained the genetic sequences of the RBD (636 nt), followed by complete transmembrane domain (TM), and cytoplasmic tail (CT) of the NDV haemagglutinin-neuraminidase (HN) gene. Second, the S1-F transcriptional cassette (2441 nt), which contained the genetic sequence of the S1 subunit (2043 nt), taken from the S gene (3822 nt). This sequence was fused with the TM and CT of the fusion (F) gene (Fig. 8A). These TM and CT gene sequences of HN and F genes were obtained from the pFLC-LS1 plasmid. Both transcriptional cassettes were flanked with specific gene-end (GE) and gene-start (GS) transcriptional signals of the paramyxovirus genome^57^. Further, these cassettes, flanked with restriction sites of *BbvCI,* were chemically synthesized and were subsequently cloned into plasmid pUC57 by GenScript (Piscataway, NJ, USA). These plasmids were purified and DNA extracted using QIAGEN Plasmid Midi Kit (100), according to the manufacturer’s instructions.

The pFLC-LS1 plasmid, containing a unique *BbvCI* site, was digested with *BbvCI* enzyme to obtain the linearized plasmid. Both the HN-RBD and S1-F transcriptional cassettes were digested with the same enzyme and inserted into the P/M junction of the pFLC-LS1 to be expressed as a separate mRNA. The resulting plasmids were designated as pFLC-LS1-HN-RBD (20,315 nt) and pFLC-LS1-S1-F (21,743 nt) (Fig. 8B).

#### Recovery of the rLS1-HN-RBD and rLS1-S1-F virus

Briefly, the rLS1-HN-RBD and rLS1-S1-F viruses were recovered by co-transfection of a full-length plasmid complementary DNA (cDNA) of each construct, pFLC-LS1-S1-F or pFLC-LS1-HN-RBD respectively, as described previously^56^. The recovered viruses were injected into the allantoic cavities of 9-day-old specific pathogen-free (SPF) embryonated chicken eggs (Charles River, Norwich, CT, USA). After incubation for four days at 37 °C, the allantoic fluids containing the recovered virus were harvested, clarified, aliquoted and stored at − 80 °C. The presence and recovery of viruses were confirmed by hemagglutination (HA) assays using 1% chicken red blood cells. The identity of the recombinant viruses was confirmed by reverse transcription-polymerase chain reaction (RT-PCR) and by Sanger sequencing, as described before^48^.

#### Indirect immunofluorescence assay (IFA)

To examine the SARS-CoV-2 RBD and S1 subunit proteins expression, Vero-E6 cells were infected with the recombinant rLS1-HN-RBD, rLS1-S1-F or rLS1 viruses at a multiplicity of infection (MOI) of 0.5. After 48 hours post-infection (hpi), the cells were fixed with 4% paraformaldehyde for 25 minutes (min), and then the monolayer was washed three times with Dulbecco’s phosphate-buffered saline (DPBS) and permeabilized with Triton 0.1% X-100 for 15 min at room temperature (RT). After washing with the cells with DPBS, the monolayer was incubated with the rabbit polyclonal antibody specific to SARS-CoV-2 RBD protein (1:200) (Sino Biological, Beijing, China), and a chicken antiserum specific to Newcastle disease virus (1:200) (Charles River, Norwich, CT, USA) for 1.5 h at RT. Afterwards, the monolayer was incubated with Donkey Anti-Rabbit IgG H&L-Alexa Fluor® 594 (1:250) and Goat Anti- Chicken IgY H&L-Alexa Fluor® 488 (1:1000) (Abcam, Cambridge, MA, USA) for 60 min at RT. Finally, the cells were developed with 4′,6-diamidino-2-phenylindole (DAPI) for 5 min and observed using an ObserverA1 fluorescence microscope (Carl Zeiss, Germany). Digital images were taken at 400× magnification and processed with the AxioCam MRc5 camera (Carl Zeiss, Germany).

#### Western blotting analysis

To evaluate the SARS-CoV-2 RBD and S1 subunit proteins expression, Vero-E6 cells were infected with the recombinant viruses mentioned above at an MOI of 1.0. At 48 hpi, the cells were harvested, lysed, and analyzed by Western blot. Additionally, to verify the incorporation of the RBD and S1 subunit proteins into rLS1-HN-RBD and rLS1-S1-F viruses, viral particles from allantoic fluid of SPF chicken embryonated eggs infected with the recombinant viruses and rLS1, were concentrated by ultracentrifugation (Ultracentrifuge, Beckman Coulter) at 18,000 revolutions per minute (rpm) at 4 °C, and partially purified on a 25% sucrose cushion. Western blot analysis was carried out using partially purified viruses from allantoic fluid and lysate from infected cells, using a rabbit polyclonal antibody specific to SARS-CoV-2 RBD protein (Sino Biological, Beijing, China) (2/5000) as the primary antibody and anti-Rabbit IgG conjugated to HRP (GenScript, Piscataway, NJ, USA) (2/5000) as a secondary antibody. For the detection of beta-actin as a loading control in lysate cells, using mouse monoclonal antibody to beta Actin (Abcam, Cambridge, MA, USA) (5/5000) as the primary antibody and goat anti-mouse IgG conjugated to HRP (GenScript, Piscataway, NJ, USA) (3/5000) as the secondary antibody. The protein expression was visualized with a CCD camera Azure c600 imaging system (Azure Biosystems, Dublin, USA).

#### Detection of RBD and S1 subunit proteins on the viral surface by flow cytometry

To determine the presence of RBD on the viral surface of rLS1-HN-RBD, and the presence of the S1 subunit on rLS1-S1-F viruses, virion particles were purified with a 25% sucrose cushion. Vero-E6 cells were harvested and washed with DPBS with 5% FBS. Approximately, 1 × 10^6^ cells were blocked with DPBS with 5% of naïve mouse serum for 30 min at 37 °C. Then, the cells were incubated with rLS1 (0.36 mg/mL), rLS1-S1-F (0.09 mg/mL) or rLS1-HN-RBD (0.2 mg/mL) purified viruses for 30 min at 37 °C. To remove the residual viral particles not attached to Vero-E6, the cells were washed with DPBS and 5% FBS twice. Subsequently, cells were incubated with rabbit monoclonal antibody anti-SARS- CoV-2 S1 (1:200) (Sino Biological, Beijing, China) as primary antibody for 1h at 37 °C, followed by goat anti-rabbit IgG Alexa Fluor® 488 (1:200) (Abcam, Cambridge, MA, USA) as secondary antibody.

Finally, the cells were analyzed in FACS Canto II (BD Biosciences, USA) flow cytometer. The data obtained were analyzed using the software FlowJo v.10.6 (BD Biosciences, USA), where the percentage of positive cells was taken to indicate detection of the SARS-CoV-2 S1 subunit or RBD on the viral surface of viruses bound to Vero-E6.

#### Detection of RBD and S1 subunit genes by RT-PCR

For the detection of rLS1-HN-RBD and rLS1-S1-F recombinant virus, viral RNA was extracted from allantoic fluid stocks using the QIAamp MinElute Virus Spin kit. The cDNA was generated from RNA using ProtoScript II cDNA Synthesis kit (New England Biolabs, USA), according to the manufacturer’s instructions. The cDNA was amplified using the high-fidelity DNA polymerase Master Mix Q5 (New England Biolabs, USA), with the primers NDV-3LS1- 2020-F1 (5′-GATCATGTCACGCCCAATGC-3′) and NDV-3LS1-2020-R1 (5′-GCATCGCAGCGGAAAGTAAC-3′) to amplify the complete inserts. The thermal cycling protocol comprised an initial denaturation step at 98 °C for 30 seconds (s), followed by 35 cycles of 98 °C for 10 s, 72 °C for 20 s, 72 °C for 30 s for the detection of rLS1-HN-RBD**,** and 40 s for the detection of rLS1-S1-F. The final extension step was at 72 °C for 2 min.

#### In vitro replication properties of the rLS1-HN-RBD and rLS1-S1-F viruses, plaque assay, and pathogenicity

We compared the infectivity and growth properties between the rLS1-HN-RBD, rLS1-S1-F, and rLS1 viruses. DF-1 cells were seeded at 70% confluence in 12-well plates and infected with rLS1-HN-RBD, rLS1-S1-F, and rLS1 viruses at an MOI of 0.5. Cells were maintained with DMEM containing 1% FBS and 5% allantoic fluid and incubated at 37 °C with 5% CO_2_. Supernatants of the infected cells were collected at 12, 24, 36, 48, 60, and 72 hpi and kept at − 80 °C. The titers of each collected supernatant were determined using plaque assay, as described previously^56^. These experiments were repeated at 3 specific time points. In addition, the morphology and size of the plaques of the two recombinant viruses were compared with those formed with rLS1 infection. To determine the pathogenicity, the viruses were evaluated by the Mean Death Time (MDT) and Intracerebral Pathogenicity Index (IPIC) assays in 10-day-old SPF embryonated chicken eggs and one day old SPF chickens (Charles River Avian Vaccine Services, Norwich, CT, USA), respectively, using standard procedures^58^.

#### Genetic stability of the rLS1-HN-RBD and rLS1-S1-F viruses

The genetic stability of the recombinant viruses across multiples passages was evaluated on 9-day-old SPF embryonated chicken eggs, the viral RNA was extracted from purified viruses of the 3rd and 6th passage, and the presence of the gene inserts was confirmed by RT-PCR using specific primers. The expression of the SARS-CoV-2 S1 subunit and RBD inserts was also evaluated using purified viruses of the 3rd and 6th passage by Western blotting.

#### Preparation and stability of the lyophilized vaccine

To check the stability of the lyophilized vaccine, the rLS1-RBD-HN and rLS1-S1-F viruses were separately inoculated into the allantoic cavities of 9–11-day-old SPF embryonated chicken eggs. After four days of incubation at 37 °C, the allantoic fluids were harvested, clarified, and filtered using 0.22 µm filters. The presence of the viruses in allantoic fluid was detected and confirmed by HA. Finally, the allantoic fluids containing the rLS1-RBD-HN, rLS1-S1-F, and the mixture of both viruses (equimolar 1:1), were placed in vials (2 mL/vial) and lyophilized using an MX5356 lyophilizer (Millrock Technology). The lyophilized vaccine of the mixture of rLS1-HN-RBD and rLS1-S1-F viruses were stored at 4 °C and were evaluated by plaque assay, HA, and Western blot assays on days 1, 30, and 50 after lyophilization. The lyophilized vaccines were used in the following in vivo tests in hamsters.

### Immunogenicity in hamsters

Forty-eight Golden Syrian hamsters, weighing between 120 and 140 g, were divided into 4 groups (*n* = 12 per group): group 1 (rLS1-HN-RBD), group 2 (rLS1-S1-F), group 3 (rLS1-HN-RBD/rLS1-S1-F), and the unvaccinated control group 4, were intranasally immunized with 5 × 10^6^ plaque-forming units (PFUs)/hamster (40 µL volume) following a prime-boost regimen with a two-week interval. Immunized hamsters were bled immediately before the boost and fifteen days post-boost (at days 15 and 30 respectively), to measure the SARS-CoV-2 RBD and S specific serum IgG antibody by indirect enzyme-linked immunosorbent assay (ELISA), neutralizing antibody (nAbs) titers using a surrogate Virus Neutralization Test (sVNT), and to perform Plaque Reduction Neutralization Test (PRNT) against SARS-CoV-2 virus (Fig. 5A).

#### Indirect enzyme-linked immunosorbent assay (ELISA) to determine IgG levels in serum

Immunized hamsters were bled on days 15 and 30 after immunization. All sera were isolated by centrifugation at 2500 rpm for 5 min. To perform the assay, Nunc MaxiSorp 96-well flat-bottom plates were coated with 100 µL of SARS-CoV-2 RBD and S1 subunit purified proteins (1 µg/mL) (GenScript, Piscataway, NJ, USA), dissolved in carbonate-bicarbonate buffer (pH 9.6) and incubated at 4 °C overnight. After coating the plates, standard ELISA protocol was followed as described earlier^48^.

#### Neutralization tests using SARS-CoV-2 surrogate virus

Serum samples were processed to evaluate nAbs titers against SARS-CoV-2. All neutralization assays were performed with the sVNT, (GenScript, Piscataway, NJ, USA), following the manufacturer’s instructions. The positive and negative cut-offs for SARS-CoV-2 nAbs detection were interpreted as inhibition rate, as follows: *positive*, if ≥ 20% (neutralizing antibody detected), and *negative*, if < 20% (neutralizing antibody no detectable).

#### Plaque reduction neutralization test (PRNT)

SARS-CoV-2 (28549) was isolated from a nasopharyngeal swab sample collected from a patient with confirmed SARS-CoV-2 infection in April 2020 in Lima, Peru. The identity of the virus was confirmed by whole genome sequencing. Virus isolation was performed using Vero 81 cells maintained in EMEM supplemented with 10% FBS, 100 IU/mL of penicillin and 100 µg/mL streptomycin and cultured at 37 °C in an incubator with humidified atmosphere at 5% CO_2_. The sample was filtered through a 0.22 µm pore membrane. Then, 100 µL were used to inoculate Vero 81 cells. Cells were observed daily to detect the appearance of any cytopathic effect and virus was collected for confirmation. The virus was propagated in Vero 81 cell culture for viral stock production and stored at − 80 °C and titer determined by PFU.

Hamster serum samples collected at day 30 after immunization were pooled and then heat-inactivated at 56 °C for 30 min. Then after two-fold serial dilutions, serum samples were mixed and incubated with 40–50 PFUs of SARS-CoV-2 (28549) for 1 h at 37 °C in 5% CO_2_. These serum SARS-CoV-2 mixtures were added to Vero-E6 cells (in a 24-well plate) and incubated at 37 °C for 1 h. After absorption, the serum-virus mixtures were removed, and a liquid overlay medium (L-OM) comprising 0.75% carboxymethylcellulose (CMC) (Sigma-Aldrich) supplemented with 2% FBS was added to the monolayers and set to incubate at 37 °C/5% CO_2_ for 5 days. The plates were fixed and stained with 10% formaldehyde and 0.5% crystal violet solution^59^. Each serum sample was tested in duplicate. The plaques were enumerated for the calculation of PRNT50, considered the gold standard method^60^.

#### Cytokines quantification by qPCR

Fifteen days post-immunization, spleens were collected from hamsters immunized with the different recombinant viruses and stored in RNA-later reagent at 4 °C overnight and then at − 80 °C. RNA was extracted with RNeasy Mini kit, converted to cDNA with ProtoScript® II cDNA Synthesis kit, and stored at − 20 °C until analysis. Interferon-gamma (IFNγ), Tumor Necrosis Factor-Alpha (TNF-α) and interleukin-10 (IL-10), and reference gene β-actin were evaluated with primer pairs reported previously^61–64^. Standard curves were made for all primers, obtaining acceptable efficiency and R^2^ values (data not shown). Mixes were prepared using Luna® Universal qPCR Master Mix kit (New England Biolabs), according to manufacturer's instructions. Briefly, 5 µL of the sample were used (~ 2 ng/µL cDNA) with 2–3 technical replicates. The quantitative Real-Time PCR (qPCR) experiments were done on the Rotor-Gene Q equipment (Qiagen, Hilden, Germany) and the ΔΔCT method^65^ was used for data analysis.

#### Cytokines quantification by ELISA

Fifteen days post-immunization, whole blood obtained from hamsters immunized with rLS1-HN-RBD, rLS1-S1-F, and rLS1-HN-RBD/rLS1-S1-F or allantoic fluid (mock) was centrifuged at 1000×*g* for 20 min at 4 °C to obtain the serum, which was duly aliquoted, frozen, and stored at − 80 °C until analysis. For the quantitative ELISA, several kits for the accurate quantitative detection of hamster cytokines such as, TNFα, IFNγ, IL-2, IL-4, and IL-10 were purchased from MyBioSource, Inc., San Diego, CA. Cytokine quantifications were performed following the manufacturer's instructions. The plates were read in the EON spectrophotometer (Biotek, USA) at 450 nm. The level of cytokines (pg/mL) detected in the serum of the animals vaccinated with rLS1-HN-RBD, rLS1-S1-F, and rLS1-HN-RBD/rLS1-S1-F were compared with mock vaccinated animals.

### Efficacy of the vaccines against SARS-CoV-2 challenge

Forty-eight golden Syrian hamsters, divided into 4 groups (*n* = 12): group 1 (rLS1-HN-RBD), group 2 (rLS1-S1-F), group 3 (rLS1-HN-RBD/rLS1-S1-F), and the unvaccinated control group 4, were intranasally challenged with 1 × 10^5^ PFU SARS-CoV- 2/hamster (Wuhan B.1.1 strain; Accession ID: GISAID EPI_ISL_1092347, kindly donated by the National Institute of Health, Lima, Peru) in DMEM (40 µL volume) at 45 days post-prime immunization. Four animals in each group were anesthetized and sacrificed with an overdose of 1 mL of a mixture of ketamine (100 mg), xylazine (20 mg), and atropine sulfate (1 mg) by intramuscular injection at 2, 5, and 10 days post-challenge (dpc).

The lung tissue samples (right and left lungs) were separated into two parts: (1) The right lobe was used for the pathological examination, and (2) the left lung was immediately frozen at − 80 °C until used; this lung was used for live infectious virus by viral isolation. All work and handling with SARS-CoV-2 was performed in a BSL-3 laboratory following the biosafety guidelines of INS.

#### Histopathology analysis

Lungs obtained from sacrificed hamsters at days 2, 5, and 10 post-challenge with SARS-CoV-2 were fixed in 10% buffered formalin for 48 h. Tissues were then trimmed and placed in a container for 24 h with buffered formalin. The containers with the organs were processed in an automatic tissue processor (Microm brand) conducting the following processes: dehydration, diaphanating, rinsing, and impregnation within an 8 h cycle. Organs embedded in paraffin were cut to a thickness of 5 microns (Microtome Leica RM2245 of disposable metal blades), placed in a flotation solution in a water bath and then fixed on a slide sheet, and dried in the stove (at 37 °C for 1 to 2 h). The staining was done with Hematoxylin and Eosin (H&E). The final slides stained with H&E were taken and analyzed under an AxioCam MRc5 camera and AxioScope.A1 microscope (Carl Zeiss, Germany) at a magnification of 20 and 40× by a board-certified veterinary pathologist.

#### Viral viability: culture and immunofluorescence assay (IFA)

For virus viability, 60 lung tissue samples from challenged animals were crushed and homogenized in 5 % w/v of DMEM 1% antibiotic-antimycotic and centrifuged at 10,000 rpm for 10 min at 4 °C. The supernatant was filtered with a 0.22 µm Millipore filter membrane, then 100 µL were inoculated into a confluent monolayer of the Vero 81 cells, and cultured at 37 °C in an incubator with humidified atmosphere at 5% CO_2_. The cultures were observed daily for 10 days through the inverted microscope. The IFA was performed using a polyclonal antibody against SARS-CoV-2 from COVID-19 convalescent patients, and anti-human IgG conjugated with HRP (Sigma).

#### Viral load in lung

Lung virus isolation was confirmed by quantitative reverse transcription PCR (RT-qPCR), as described previously^66^. SARS-CoV-2 viral load levels were quantified using the Allplex n-CoV-2019 RT-PCR kit (Seegene®) for SARS-CoV-2 detection. The supernatant or suspension obtained from the hamster lung homogenate was subjected to extraction and purification of total RNA and DNA by the Maxwell® RSC Viral Total Nucleic Acid Purification Kit magnetic bead kit, and the Maxwell® 16 Instrument semi-automated robot. Purified RNA from the samples was added to the RT-PCR reaction mixture of the Allplex kit and analyzed according to the kit recommendations by the CFX96 thermal cycler kit (BIORAD®). Viral copy quantification analysis was performed based on the Ct value of the SARS-CoV-2 virus N gene.

#### Animal mobil

To assess hamster’s mobility (in groups 1 to 4) post-challenge, the average velocity, average acceleration, and average displacement were calculated based on videos with a camera positioned on top of the hamsters. The videos were analyzed on days 2, 5, and 10 post-challenge.

It should be noted that the conditions of video recording (distance and focus) were kept the same; therefore, the pixels always reflect the same distance. Since hamsters do not necessarily move a lot at the border of the box, we estimated average velocity, acceleration and displacement based on any movement that took place away from the edges the box (Supplementary Figure 4). Movement along the edges of the box were excluded and we tracked movement through a 2–3 min time period. Subsequent observations were recorded only when the hamsters were in the center of the box. Tracking was carried out using the Kernelized Correlation Filter (KCF)^67^. The implementation of this tracking algorithm was developed using the OpenCV library and the Python language. The result of tracking the hamsters was a record of the positions (X and Y) of the hamster in the image. Finally, once the tracking record was obtained at the intervals of interest, the average velocity, average acceleration, and average displacement were calculated for each of the hamsters.

#### Animal weight variation

The body weight change was measured on days 2, 5, and 10 post-challenge. An additional mock group (*n* = 12) of unvaccinated and unchallenged animals outside the BSL-3 were evaluated. These measurements were used to calculate the percentage of body weight variation, compared to day 0 for each animal.

### Statistical analysis

For the statistical analysis of the weight variation in hamster groups, we used the one factor analysis of variance (ANOVA) in the statistical package Stata software v.16. For the comparison of treatments of the quantification of Cytokines, by qPCR and ELISA, we used the non-parametric Mann–Whitney–Wilcoxon test. Both tests were performed using the statistical software STATAv.16. To evaluate the statistical significance of body weight change in hamster groups, and a one-way ANOVA with multiple comparisons for all the treatments involved was performed in the software GraphPad Prism v.8.0.1. To evaluate changes in hamsters’ mobility over time, nonparametric statistics using the Mann–Whitney and Kruskal–Walls tests were used SciPy v1.5.2 package. In all analyses, *P* < 0.05% is considered statistically significant. To assess plaque reduction (%) of neutralization from the different groups of hamsters, we used two-way ANOVA and Tukey’s post hoc test using GraphPad Prism v.8.0.1.

### Patent

Peruvian patent # N33-2021/DIN has been filed for the vaccine candidates presented in this study.

## Supplementary Information

Supplementary Information.

## Data Availability

All relevant data are contained within the manuscript and the supplementary material. Additional raw data will be available upon request.
