# Supplementary material for: Intranasal vaccination of hamsters with a Newcastle disease virus vector expressing the S1 subunit protects animals against SARS-CoV-2 disease
Source: Sci Rep. 2022 Jun 20;12:10359. doi: 10.1038/s41598-022-13560-z (PMC9208357; doi:10.1038/s41598-022-13560-z)

**Supplementary Figure 1**


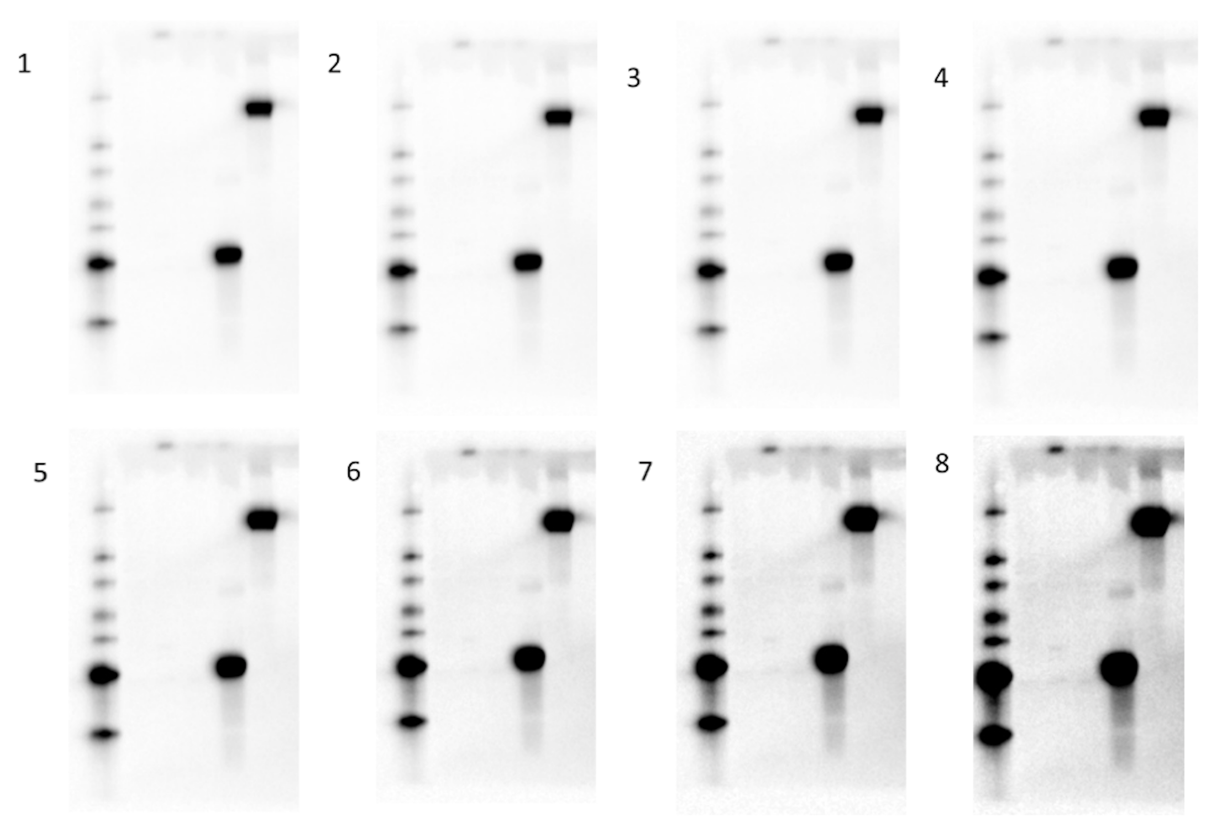


**Supplementary Figure 1.** Expression of SARS-CoV-2 RBD and S1 proteins in infected Vero-E6 cells and NDV particles. Western blot detection for the HN-RBD and S1-F proteins expression. Vero-E6 cells were infected with the rLS1, rLS1-HN-RBD, and rLS1-S1-F viruses at an MOI of 1.0. After 48 hpi, the cells were lysed and analyzed by Western blotting.

**Supplementary Figure 2**

**
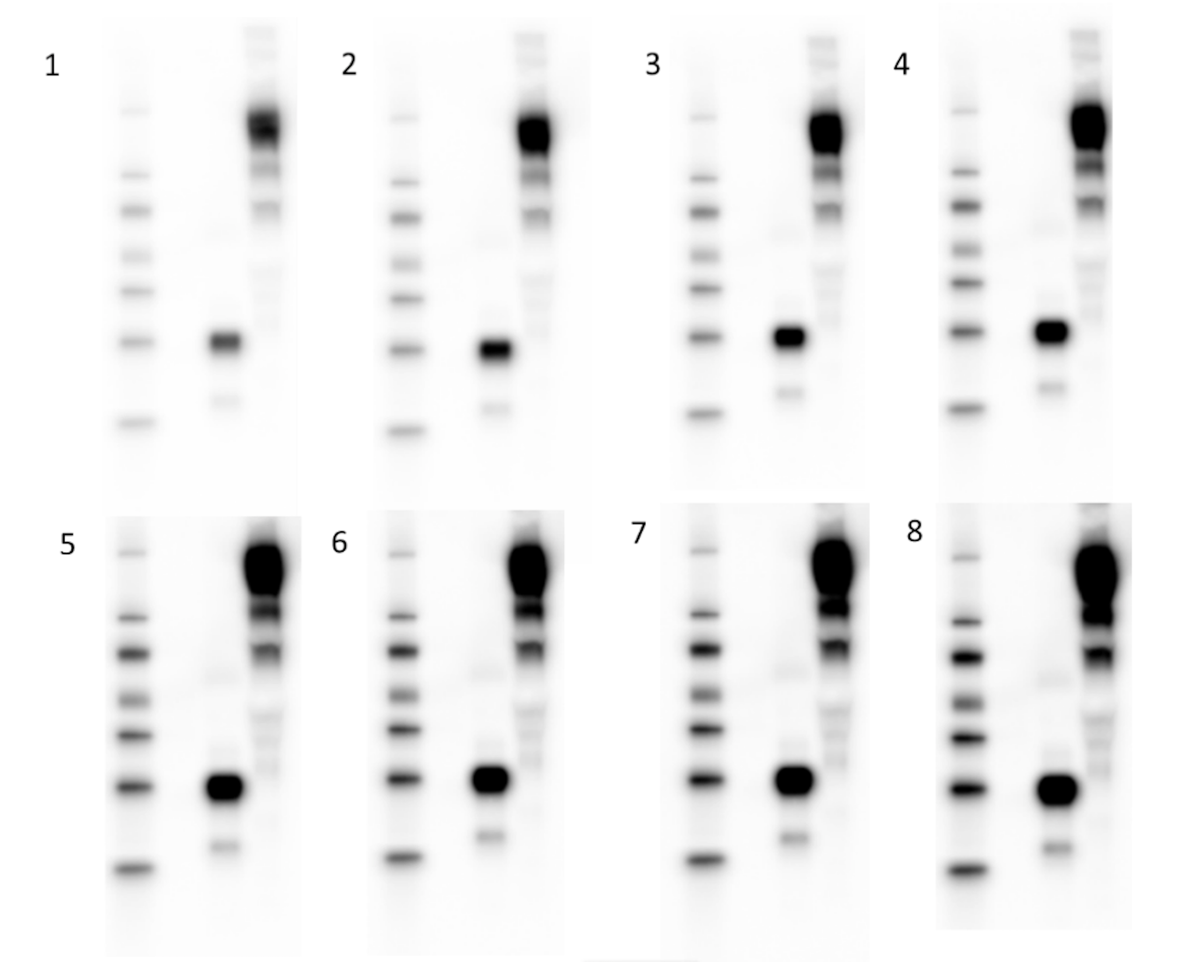
**

**Supplementary Figure 2.** Expression of SARS-CoV-2 RBD and S1 proteins in infected Vero-E6 cells and NDV particles. To verify the incorporation of the HN-RBD and S1-F proteins into rLS1-HN-RBD, and rLS1-S1-F viruses, the viral particles in allantoic fluid of infected SPF chicken embryonated eggs with the recombinant viruses and rLS1, was concentrated by ultracentrifugation, and partially purified on a 25 % sucrose cushion. Western blot analysis was carried out using partially purified viruses, using a rabbit antibody specific to SARS-CoV-2 RBD protein and Anti-rabbit IgG conjugated to HRP. The black arrow indicates the expected protein band. The gels are shown with equal running conditions. The protein expression was visualized with a CCD camera Azure c600 imaging system (Azure Biosystems, Dublin, USA). Each panel includes 8 images with multiples exposure.

**Supplementary Figure 3**

**
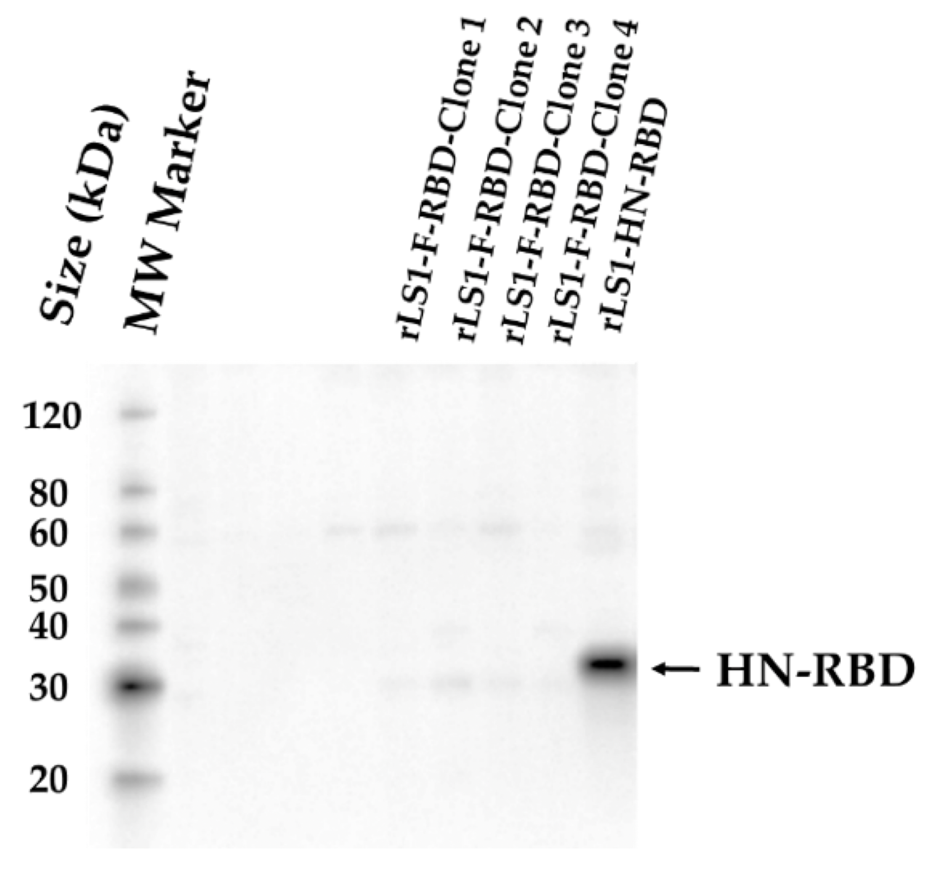
**

**Supplementary Figure 3.** Expression of SARS-CoV-2 RBD protein in infected Vero-E6 cells. Western blot detection for the RBD protein expression, Vero-E6 cells were infected with the rLS1-F-RBD (clone 1-clone 4) and rLS1-HN-RBD viruses at an MOI of 1.0. After 48 hpi, the cells were lysed and analyzed by Western blotting. Western blot analysis was carried out using lysate from infected cells, using a rabbit antibody specific to SARS-CoV-2 RBD protein and Anti-rabbit IgG conjugated to HRP. The black arrow indicates the expected protein band. The gels are shown with equal running conditions. The protein expression was visualized with a CCD camera Azure c600 imaging system (Azure Biosystems, Dublin, USA).

**Supplementary Figure 4**

**
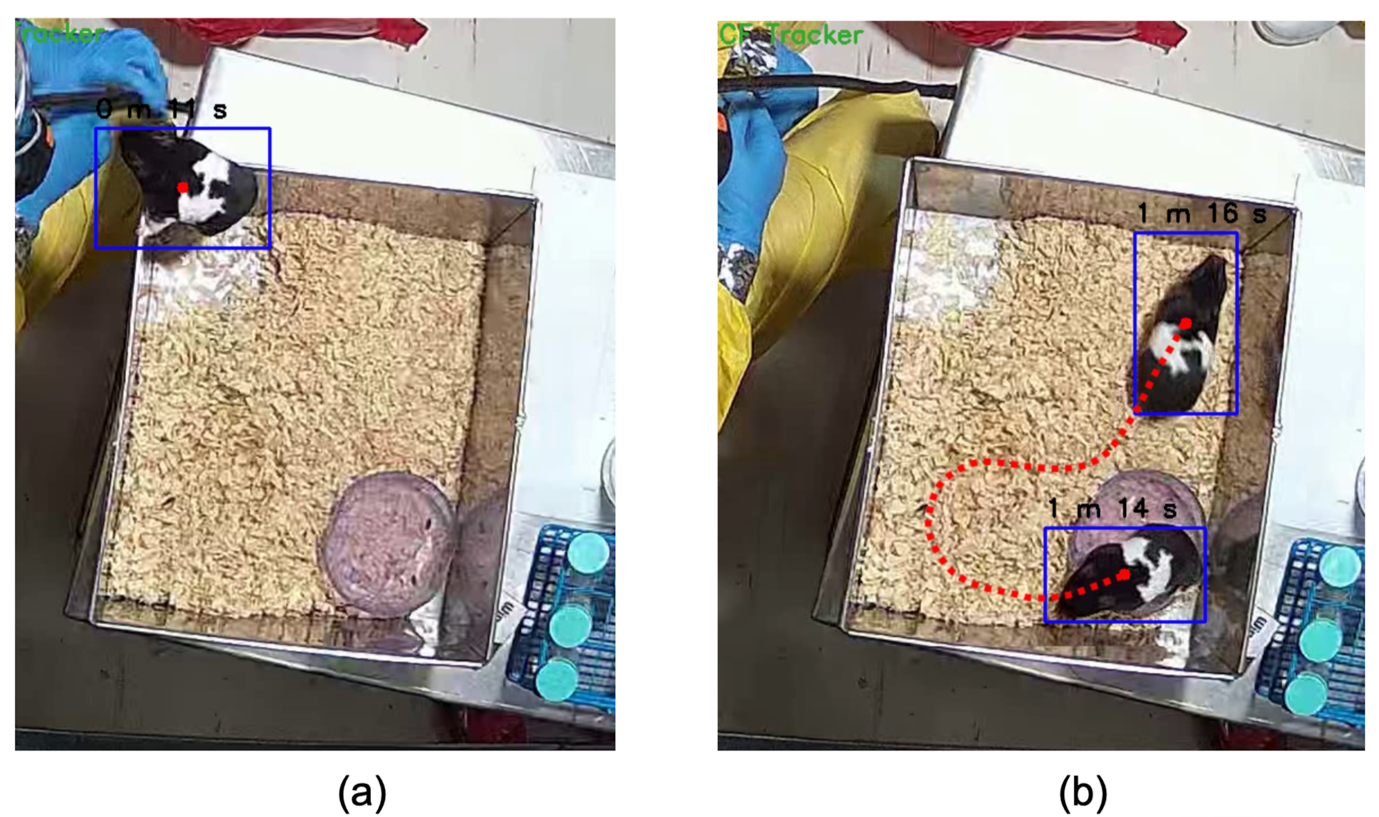
**

**Supplementary Figure 4.** Tracking of animals for body motion description. (**a**) Time intervals were not considered when the hamster was on the edges. (**b**) Time intervals were considered when the hamster remained outside the edges and had free movement.

**Supplementary Figure 5**

**
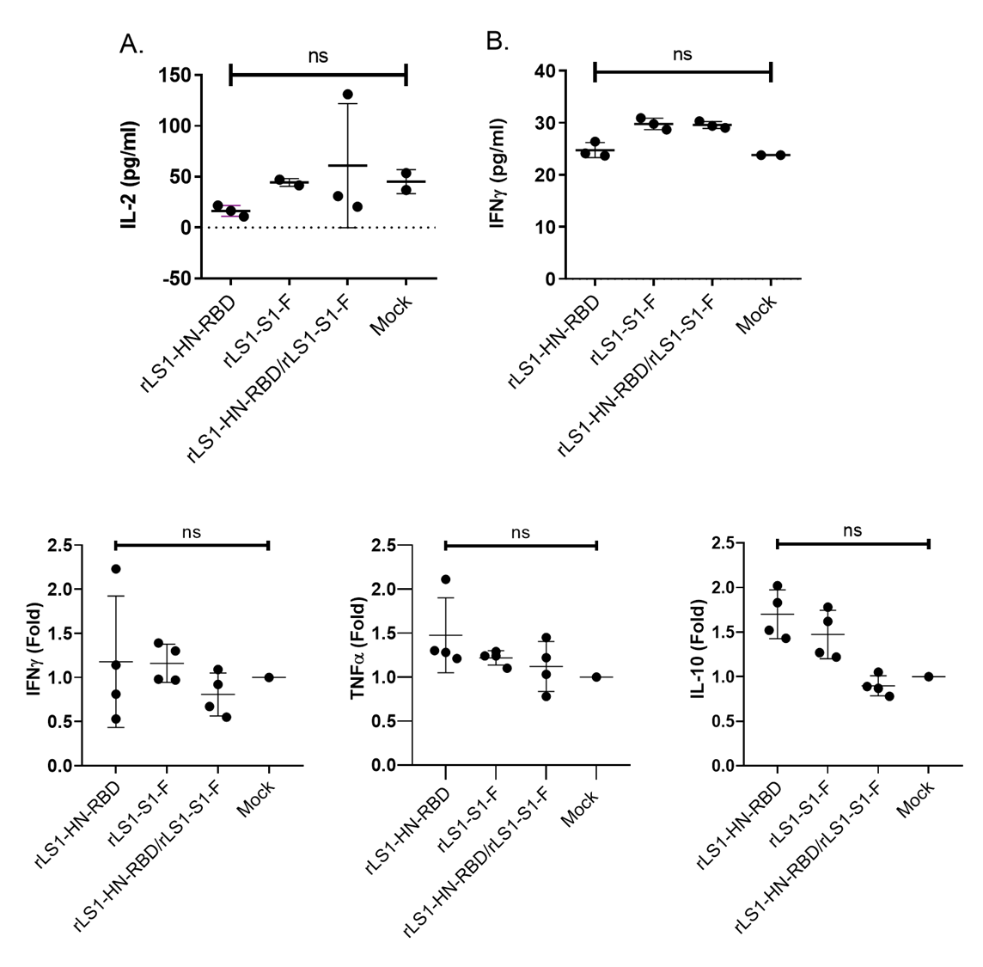
**

**Supplementary Figure 5.** Cellular immunity. These figures show cytokines measured by quantitative ELISA (pg/ml) on hamster serum immunized with rLS1-HN-RBD (*n*=3), rLS1-S1-F (*n*=3 for IFNγ, *n*=2 for IL-2), rLS1-HN-RBD/rLS1-S1-F (*n*=3) and mock (*n*=2) at 15 days post-vaccination. (**A**) IL-2 and (**B**) IFNγ, ns: not significant; P <0.05. Fold expression of cytokines by ΔΔqPCR from hamster spleens (*n*=13) vaccinated with rLS1-HN-RBD (*n*=4), rLS1-S1-F (*n*=4), rLS1-HN-RBD/ rLS1-S1-F (*n*=4), and mock (*n*=1). IFNγ (**C**), TNFα (**D**), and IL-10 (**E**), were evaluated at 15 days post-vaccination. Each individual present 3 technical replicas for GOI and 2 technical replicas for HKG, a No-RT control was included. Non- parametric Mann-Whitney-Wilcoxon test was used with Stata software v.16. P values of <0.05 were considered significant. * P <0.05, ** P <0.01, *** P <0.001, **** P<0.0001. NS, not significant.

**Supplementary Table I**

| Hamster | Cytokine measured by quantitative ELISA | | | | | | | | | | | | | | | | | | | |
| --- | --- | --- | --- | --- | --- | --- | --- | --- | --- | --- | --- | --- | --- | --- | --- | --- | --- | --- | --- | --- |
|  | TNF-α | | | | IL-2 | | | | IL-4 | | | | IL-10 | | | | IFNy | | | |
|  | (pg/mL) | O.D. | Average | CV | (pg/mL) | O.D. | Average | CV | (pg/mL) | O.D. | Average | CV | (pg/mL) | O.D. | Average | CV | (pg/mL) | O.D. | Average | CV |
| rLS1-HN-RBD ♂2 | 51.51 | 0.274 | 0.3345 | 0.26 | 21.57 | 0.085 | 0.093 | 0.12 | 0 | 0.085 | 0.093 | 0.12 | 0 | 0.05 | 0.05 | 0 | 26.4 | 0.372 | 0.369 | 0.01 |
|  |  | 0.395 |  |  |  | 0.101 |  |  |  | 0.101 |  |  |  | 0.05 |  |  |  | 0.365 |  |  |
| rLS1-HN-RBD ♂3 | 56.27 | 0.438 | 0.4245 | 0.04 | 16.35 | 0.086 | 0.081 | 0.09 | 0 | 0.086 | 0.081 | 0.09 | 0 | 0.05 | 0.05 | 0 | 24.14 | 0.308 | 0.31 | 0.01 |
|  |  | 0.411 |  |  |  | 0.076 |  |  |  | 0.076 |  |  |  | 0.05 |  |  |  | 0.311 |  |  |
| rLS1-HN-RBD ♂4 | 58.18 | 0.402 | 0.4565 | 0.17 | 10.92 | 0.066 | 0.069 | 0.05 | 0 | 0.066 | 0.069 | 0.05 | 0 | 0.05 | 0.05 | 0 | 23.7 | 0.298 | 0.298 | 0 |
|  |  | 0.511 |  |  |  | 0.071 |  |  |  | 0.071 |  |  |  | 0.05 |  |  |  | 0.297 |  |  |
| rLS1-S1-F ♂2 | - | - | - | - | - | - | - | - | - | - | - | - | - | - | - | - | 30.9 | 0.473 | 0.472 | 0 |
|  |  | - |  | - |  | - |  | - |  | - |  | - |  | - |  | - |  | 0.471 |  |  |
| rLS1-S1-F ♂3 | - | - | - | - | 47 | 0.142 | 0.152 | 0.09 | - | - | - | - | - | - | - | - | 29.77 | 0.448 | 0.448 | 0 |
|  |  | - |  | - |  | 0.161 |  |  |  | - |  | - |  | - |  | - |  | 0.447 |  |  |
| rLS1-S1-F ♂4 | - | - | - | - | 41.57 | 0.131 | 0.139 | 0.08 | - | - | - | - | - | - | - | - | 28.7 | 0.432 | 0.424 | 0.03 |
|  |  | - |  | - |  | 0.147 |  |  |  | - |  | - |  | - |  | - |  | 0.415 |  |  |
| rLS1-HN-RBD/ rLS1-S1-F ♂2 | 54.33 | 0.38 | 0.3895 | 0.03 | 131.13 | 0.343 | 0.345 | 0.01 | 0 | 0.343 | 0.345 | 0.01 | 0 | 0.062 | 0.06 | 0.05 | 29.41 | 0.442 | 0.44 | 0.01 |
|  |  | 0.399 |  |  |  | 0.347 |  |  |  | 0.347 |  |  |  | 0.058 |  |  |  | 0.437 |  |  |
| rLS1-HN-RBD/ rLS1-S1-F ♂3 | 53.07 | 0.35 | 0.365 | 0.06 | 20.48 | 0.084 | 0.091 | 0.1 | - | - | - | - | - | - | - | - | 29.03 | 0.434 | 0.431 | 0.01 |
|  |  | 0.38 |  |  |  | 0.097 |  |  |  | - |  | - |  | - |  | - |  | 0.428 |  |  |
| rLS1-HN-RBD/ rLS1-S1-F ♂4 | 53.28 | 0.32 | 0.365 | 0.17 | 30.92 | 0.1 | 0.115 | 0.18 | - | - | - | - | - | - | - | - | 30.32 | 0.45 | 0.46 | 0.03 |
|  |  | 0.41 |  |  |  | 0.129 |  |  |  | - |  | - |  | - |  | - |  | 0.469 |  |  |
| Mock ♂3 | - | - | - | - | 53.52 | 0.168 | 0.167 | 0.01 | - | - | - | - | - | - | - | - | 23.79 | 0.304 | 0.3 | 0.02 |
|  |  | - |  | - |  | 0.165 |  |  |  | - |  | - |  | - |  | - |  | 0.296 |  |  |
| Mock ♀4 | - | - | - | - | 36.79 | 0.129 | 0.128 | 0.01 | - | - | - | - | - | - | - | - | 23.79 | 0.298 | 0.3 | 0.01 |
|  |  | - |  | - |  | 0.127 |  |  |  | - |  | - |  | - |  | - |  | 0.302 |  |  |

**Supplementary Table I.** Quantification of cytokines by ELISA on vaccinated and control animals. ( - : The test was not done, due to lack of sample)

**Supplementary Table II**

|  |  |  | IFA |
| --- | --- | --- | --- |
|  | Sex: (M)ale / (F)emale | Type of vaccine |  |
| 2 days after challenge | M | Placebo | Positive |
|  | M | Placebo | Positive |
|  | F | Placebo | Positive |
|  | F | Placebo | Positive |
|  | F | NDV-RBD | Positive |
|  | M | NDV-RBD | Positive |
|  | M | NDV-RBD | Positive |
|  | M | NDV-RBD | Positive |
|  | F | NDV-S1 | Negative |
|  | M | NDV-S1 | Negative |
|  | M | NDV-S1 | +/- |
|  | F | NDV-S1 | Positive |
|  | F | NDV-S1 + NDV-RBD | Negative |
|  | F | NDV-S1 + NDV-RBD | Negative |
|  | F | NDV-S1 + NDV-RBD | Negative |
|  | M | NDV-S1 + NDV-RBD | Positive |
| 5 days after challenge | M | Placebo | +/- |
|  | M | Placebo | Positive |
|  | F | Placebo | Positive |
|  | F | Placebo | Positive |
|  | F | NDV-RBD | Positive |
|  | M | NDV-RBD | Positive |
|  | M | NDV-RBD | Positive |
|  | M | NDV-RBD | Positive |
|  | F | NDV-S1 | Negative |
|  | M | NDV-S1 | Negative |
|  | M | NDV-S1 | Negative |
|  | F | NDV-S1 | Negative |
|  | F | NDV-S1 + NDV-RBD | Negative |
|  | F | NDV-S1 + NDV-RBD | Negative |
|  | F | NDV-S1 + NDV-RBD | +/- |
|  | M | NDV-S1 + NDV-RBD | Negative |
| 10 days after challenge | M | Placebo | Negative |
|  | M | Placebo | Negative |
|  | F | Placebo | Negative |
|  | F | Placebo | +/- |
|  | F | NDV-RBD | +/- |
|  | M | NDV-RBD | Negative |
|  | M | NDV-RBD | Negative |
|  | M | NDV-RBD | Negative |
|  | F | NDV-S1 | Negative |
|  | F | NDV-S1 | Negative |
|  | M | NDV-S1 | Negative |
|  | M | NDV-S1 | Negative |
|  | M | NDV-S1 + NDV-RBD | Negative |
|  | F | NDV-S1 + NDV-RBD | Negative |
|  | F | NDV-S1 + NDV-RBD | +/- |
|  | F | NDV-S1 + NDV-RBD | +/- |

**Supplementary Table II.** Evaluation of SARS-CoV-2 presence by IFA in lung tissue of hamsters during the challenge. Vaccinated and placebo animals were analyzed.

NDV-RBD: rLS1-HN-RBD

NDV-S1: rLS1-S1-F

NDV-S1 + NDV-RBD: rLS1-S1-F/rLS1-HN-RBD

**Images showing full length membranes, with membranes edges visible**

Figure 1


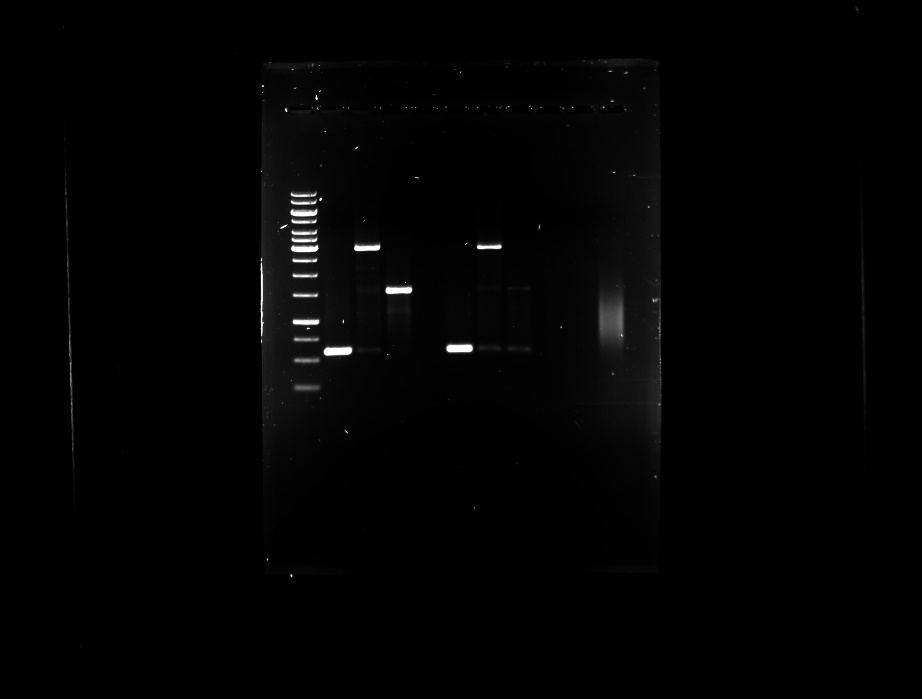


Figure 1. The insertion of the expression cassette into the non-coding region between the P/M genes of NDV genome was verified by RT-PCR using the primers NDV-3LS1-2020-F1 and NDV-3LS1-2020-R1.

Figure 2


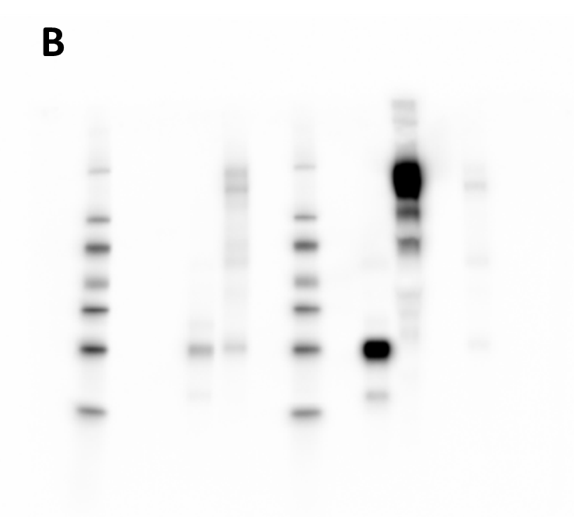


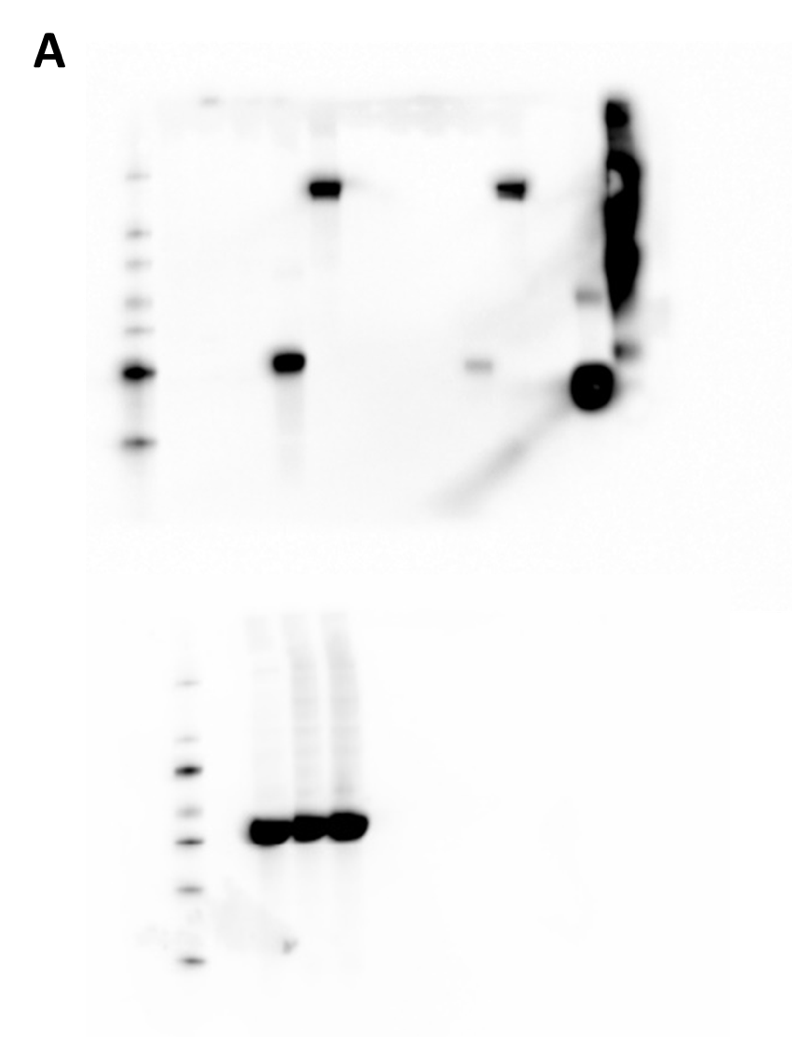


Figure 2. Expression of SARS-CoV-2 RBD and S1 proteins in infected Vero-E6 cells and NDV particles. (A) Western blot detection for the HN-RBD and S1-F proteins expression. Vero-E6 cells were infected with the rLS1, rLS1-HN-RBD, and rLS1-S1-F viruses at an MOI of 1.0. After 48 hpi, the cells were lysed and analyzed by Western blotting. (B) To verify the incorporation of the HN-RBD and S1-F proteins into rLS1-HN-RBD, and rLS1-S1-F viruses, the viral particles in allantoic fluid of infected SPF chicken embryonated eggs with the recombinant viruses and rLS1, was concentrated by ultracentrifugation, and partially purified on a 25 % sucrose cushion. Western blot analysis was carried out using partially purified viruses and lysate from infected cells, using a rabbit antibody specific to SARS-CoV-2 RBD protein and Anti Rabbit IgG conjugated to HRP. The beta-actin protein was used as a loading control in lysate cells.

Figure 3


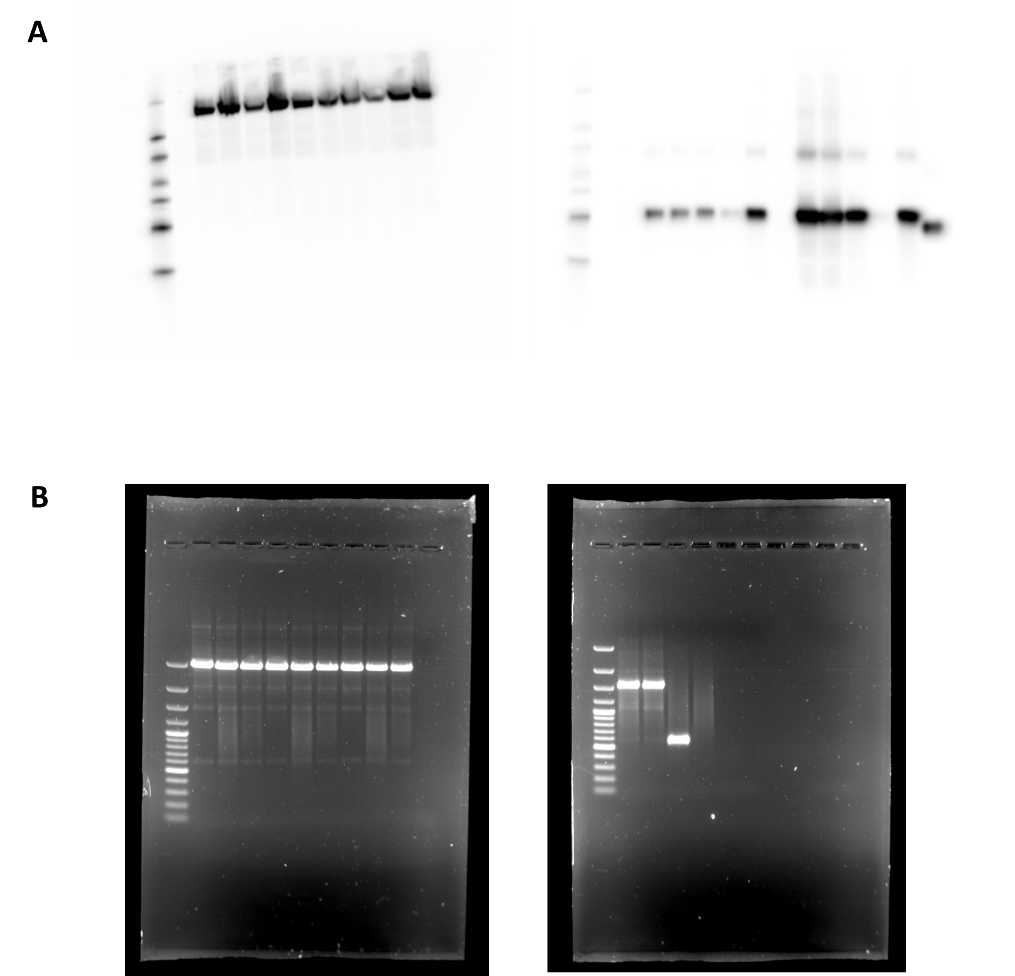


Figure 3. Genetic stability of the recombinants. The genetic stability of the rLS1-HN-RBD, and rLS1-S1-F viruses was evaluated at the 3rd and 6th passages by (A) Western blot analysis using a rabbit polyclonal antibody specific to SARS-CoV-2 RBD protein. and (B) RT-PCR using the primers NDV-3LS1-2020-F1 and NDV-3LS1-2020-R1 to amplify the complete inserts. P3: 3rd passage, P6: 6th passage.

Figure 4


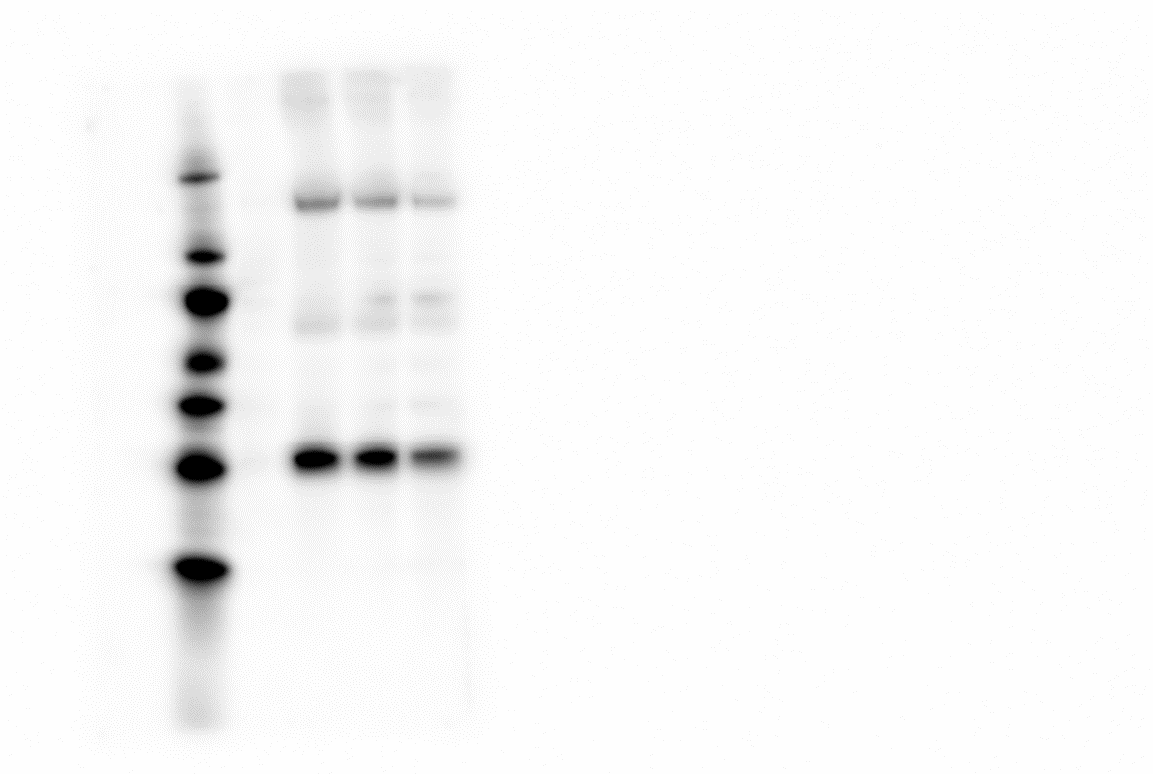


Figure 4. Stability of the lyophilized NDV vaccine. The expression of S1-F and HN-RBD proteins in Vero-E6 cells infected with the lyophilized NDV vaccine was confirmed at day 1, 30, and 50 days post-lyophilization by Western blot assay using a rabbit polyclonal antibody specific to SARS-CoV-2 RBD protein.

**Images with multiple exposures corresponding to figure 1, figure 2 (A, B), figure 3 (A, B), and figure 4.**

Figure 1


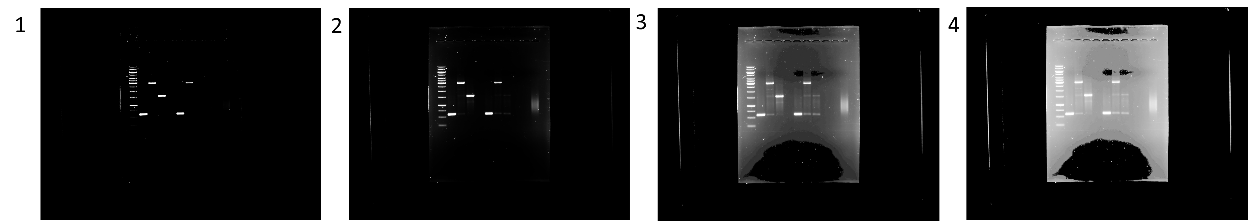


Figure 2


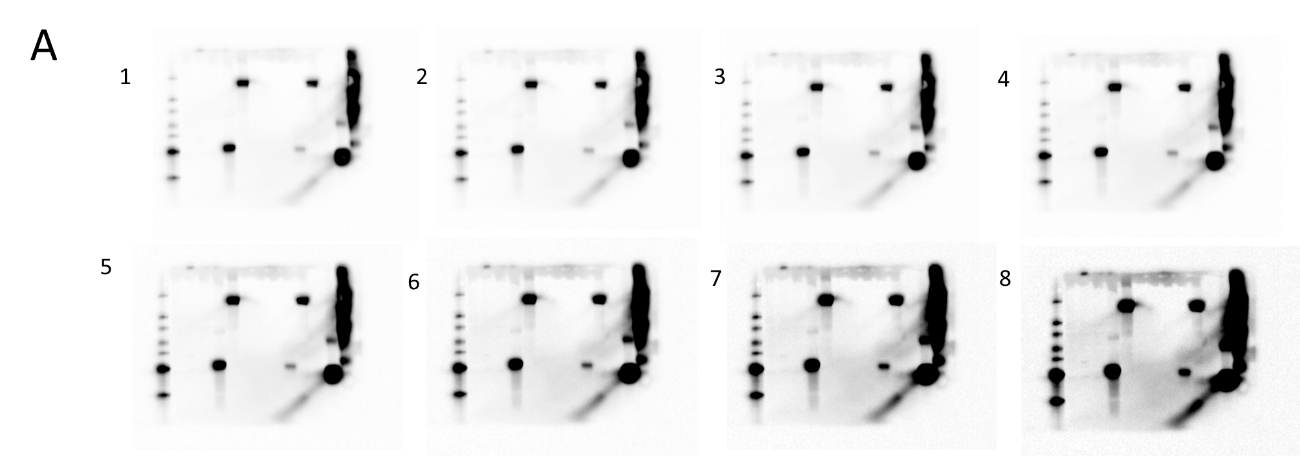


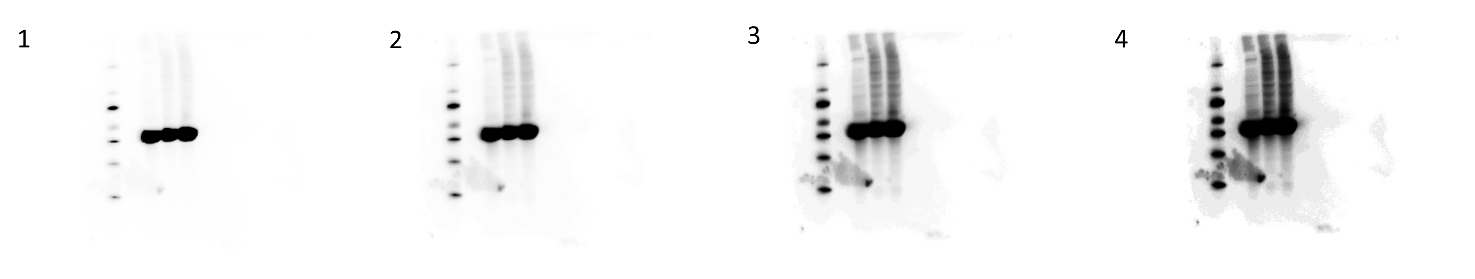


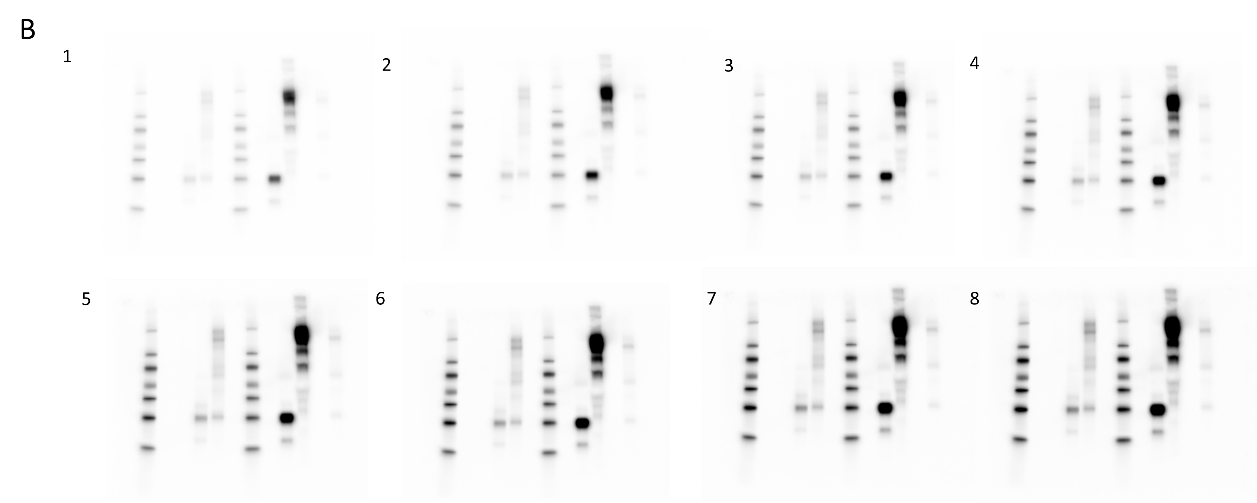


## Figure 3

## **
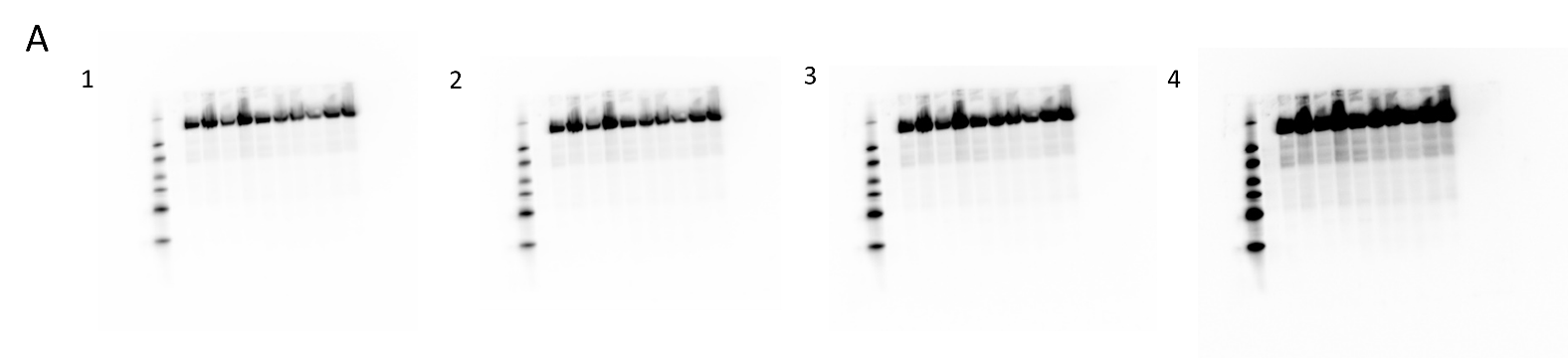
**

## **
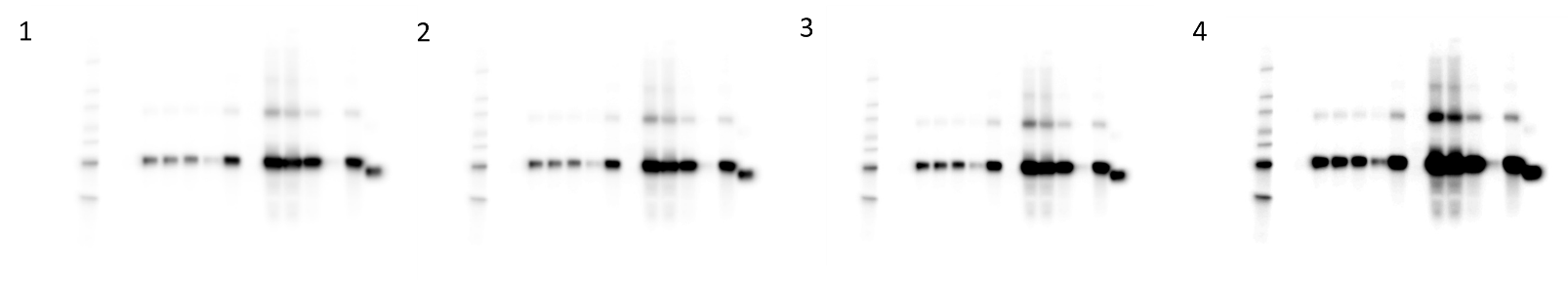
**

##
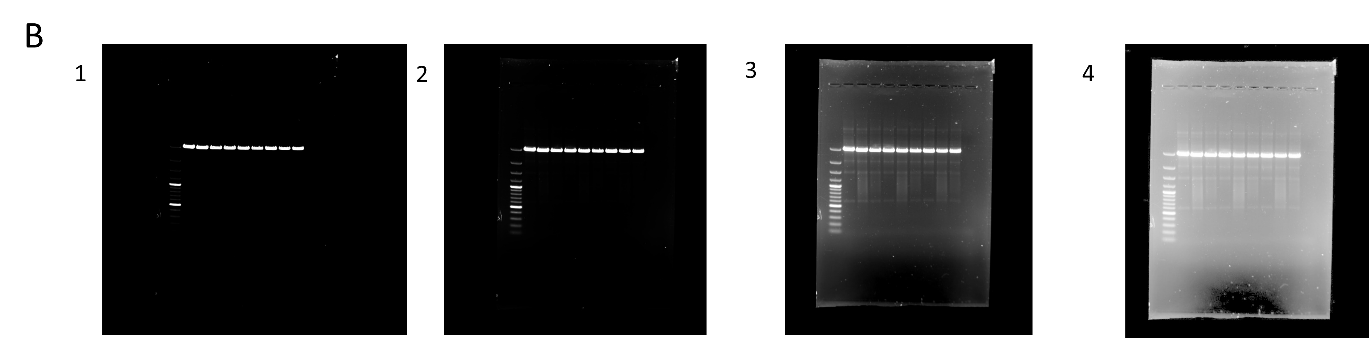


## **
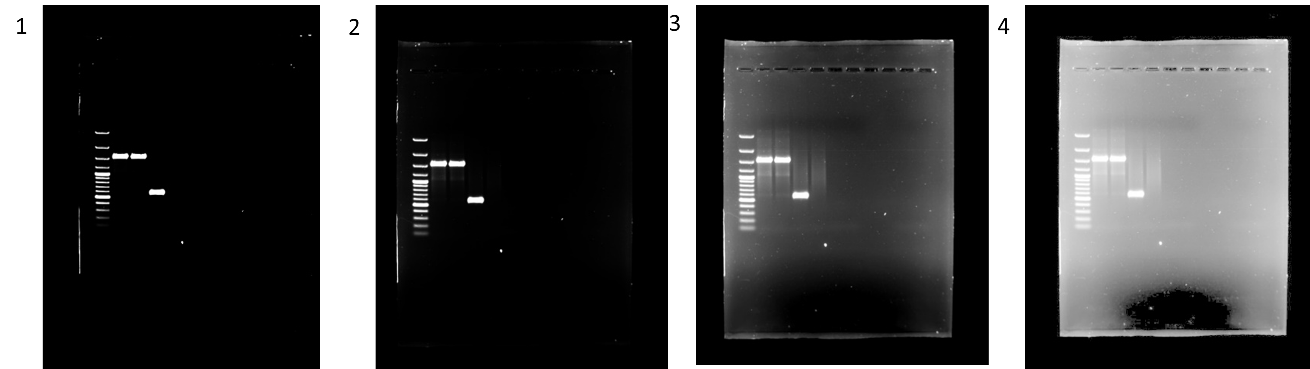
**

Figure 4


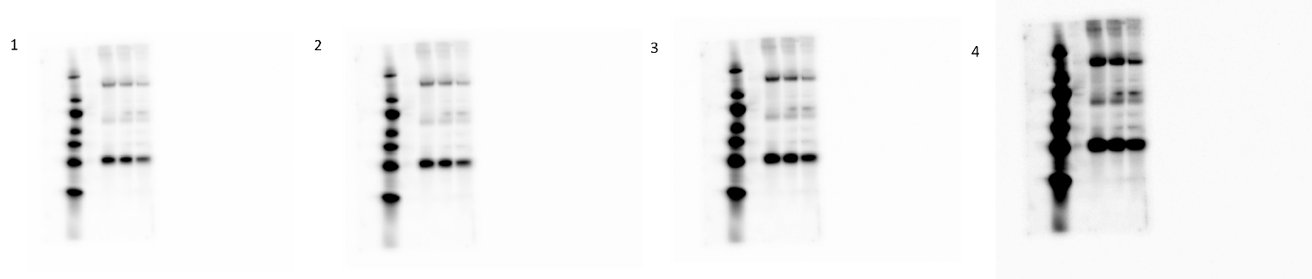

Supplement: Supplementary file 1 — Supplementary Information. [file 41598_2022_13560_MOESM1_ESM.docx]
